# Complex Factors and Challenges that Affect the Pharmacology, Safety and Efficacy of Nanocarrier Drug Delivery Systems

**DOI:** 10.3390/pharmaceutics13010114

**Published:** 2021-01-17

**Authors:** Joseph A. Piscatelli, Jisun Ban, Andrew T. Lucas, William C. Zamboni

**Affiliations:** 1 UNC Eshelman School of Pharmacy, Chapel Hill, NC 27599, USA; jpiscitelli15@gmail.com (J.A.P.); jisun_ban@unc.edu (J.B.); zamboni@unc.edu (W.C.Z.); 2 Division of Pharmacotherapy and Experimental Therapeutics, UNC Eshelman School of Pharmacy, University of North Carolina at Chapel Hill, Chapel Hill, NC 27599, USA; 3 UNC Lineberger Comprehensive Cancer Center, Carolina Center of Cancer Nanotechnology Excellence, University of North Carolina at Chapel Hill, Chapel Hill, NC 27599, USA

**Keywords:** pharmacology, nanomedicines, tumor microenvironment, nanoparticles, pharmacokinetics, pharmacodynamics, mononuclear phagocyte system (MPS)

## Abstract

Major developments in nanomedicines, such as nanoparticles (NPs), nanosomes, and conjugates, have revolutionized drug delivery capabilities over the past four decades. Although nanocarrier agents provide numerous advantages (e.g., greater solubility and duration of systemic exposure) compared to their small-molecule counterparts, there is considerable inter-patient variability seen in the systemic disposition, tumor delivery and overall pharmacological effects (i.e., anti-tumor efficacy and unwanted toxicity) of NP agents. This review aims to provide a summary of fundamental factors that affect the disposition of NPs in the treatment of cancer and why they should be evaluated during preclinical and clinical development. Furthermore, this chapter will highlight some of the translational challenges associated with elements of NPs and how these issues can only be addressed by detailed and novel pharmacology studies.

## 1. Introduction

**Figure 1:**
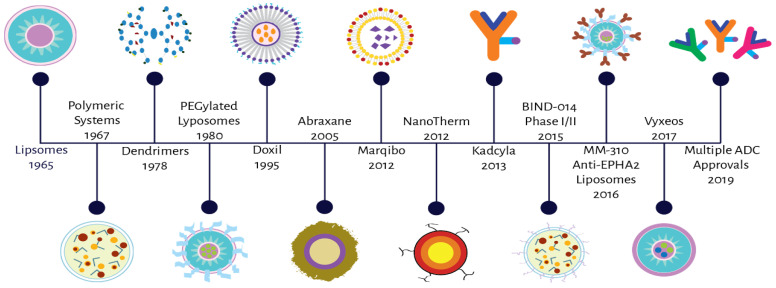
A brief history of the development of nanomedicines and other carrier-mediated agents.

Over the past three decades, advancements with carrier-mediated agents (CMAs) have led to novel drug delivery systems. Nanoparticles (NPs; such as liposomes), dendrimers, nanosomes, conjugates and antibody drug conjugates (ADCs) are just a few of these agents that are being used to uniquely target cancer cells [1]. Figure 1 highlights a timeline of the development of CMAs, showing that over the past 50 years this system of drug delivery has become more advanced and more specific. Classical NP agents have been shown to be effective in pre-clinical studies due to their ability to increase the solubility of certain anticancer agents (e.g., hydrophobic agents) and structure which allows for sustained efficacious concentrations over a longer period of time when compared to traditional small-molecule drugs [2,3]. Within the clinic, the number of agents in use is still limited, NP’s demonstrate the potential to become a life-saving drug class in cancer therapy—however, there are still concerns with these delivery systems in terms of safety and disposition. Both of these factors are hard to predict and control, leading to more questions than answers during NP development. Moreover, the focus on formulation design and manufacturing without the addition of detailed and novel analytical and pharmacology (e.g., pharmacokinetic, pharmacodynamic, and biomarker) studies has significantly hindered the translational and clinical development of NPs, especially in the treatment of cancer.

Early research into NPs started in the 1980s and has continued to improve until this day [3,4]. Although research into this drug class have been going on for more than 35 years, the number of approved NPs for therapeutic use in the clinic is low, with only 5 NPs still on the market (Table 1) [5,6,7]. Liposomes are the most common traditional NP-type agent in clinical trials right now and most of the research is being conducted in the oncology therapeutic area [3,5]. Due to the limited market approval of therapeutic CMAs, the majority of the data presented in this review will be focused on traditional nanoparticle agents used in oncology because most of the available published data. The reason that more NPs are not approved is that the pharmacology of NPs is more complex than small-molecule drugs and understanding how the body reacts to these formulations, including their disposition and safety, can be challenging as well [8,9,10]. Additionally, when testing NPs in humans, the inter-patient variability makes it difficult to predict the true nature of these formulations in terms of distribution (i.e., distribution and clearance), tumor delivery, and ultimate expected pharmacological effect (i.e., anti-tumor efficacy or unwanted toxicity).

Since it’s been four-decades of NP development and research, it would be expected that more delivery systems in this class are approved. For example, there have only been six FDA-approved liposomal NP drugs. This review discusses the various factors that affect the disposition, safety, and efficacy of classical nanocarrier NPs in the treatment of cancer. NP formulations are complex in nature and these factors influence both their pharmacokinetic (PK) and pharmacodynamic (PD) profile, which further translates into several translational challenges that come with scaling and prediction of pre-clinical data to patients. By understanding the complex pharmacology of NPs, future study designs and drug development can be improved for these complex agents.

## 2. Basic Pharmacokinetic & Analytical Considerations

### 2.1. Principal Differences in ADME Between Small-Molecule & NPs

Between small molecule and NP drugs, the pharmacokinetic principle of ADME (absorption, distribution, metabolism, and elimination) is different. Although there is a greater understanding of ADME in NPs, there are several key differences in the disposition of NPs, which makes them unique and challenging to evaluate and predict. These differences have been extensively reviewed [11,12,13,14,15], and a brief summary of key differences and aspects are noted below.

Absorption can only be measured with medications administered orally or subcutaneously (SQ). The extravascularly administered drug will enter the absorption phase after the administration and then crosses over membranes to reach the systemic circulation [16,17]. However, NPs face numerous physiological barriers with all types of the route of administrations (oral, intravenous [IV], intramuscular [IM], and SQ) [18]. In addition to those limitations, NPs loaded with cytotoxic drug molecules cannot be administered using IM or SQ due to off-target toxicity mediated by immune cells on the skin and higher risk of injection site adverse reaction [19,20,21].

The distribution between small-molecule drugs and NPs are also different, primarily due to the properties and characteristics of the carrier transporting the cytotoxic payload. Small-molecule drugs usually have a high distribution because they distribute to organs and tissues much easier than NPs and take advantage of various membrane transporters (e.g., OCT1, PgP) to cross from the blood circulation to the site of action [17,22,23]. Unlike small-molecule drugs, NPs have limited distribution due to vasculature and interstitial space within tissues, and cannot be diffused easily from the central to the peripheral compartment [16,18]. As such, vascular transcytosis and passive diffusion transporter play an important role in overall distribution, which guided by the structures of the nearby blood vessels (such as vessel fenestration size or membrane thickness) [18,22,23,24]. If vasculature is leaky enough, larger molecules can be transported into the tumors; this passive diffusion effect is discussed further in Section 3.

Small-molecule metabolism works mainly through cytochrome p450 enzymes in the liver. Once a drug is absorbed within the gastrointestinal tract, drugs are then circulated through the liver for metabolism. These drugs can then be eliminated by either renal or hepatic mechanisms [16]. NP metabolism and elimination process differ compared to traditional small-molecule drugs. While many factors responsible for the process have been identified, the mononuclear phagocyte system (MPS), which is part of the innate immune system (IIS), is believed to contribute the most to this effect. The uptake and elimination of NPs are done by numerous MPS cells such as circulating phagocytes (e.g., monocytes, dendritic cells) or tissue residing phagocytes (e.g., Kupffer cells in the liver) [16,17,18,23]. While this interaction has been documented previously, questions still exist attributed to inter-patient differences in this MPS component, and therefore, it is important that sampling should be taken into consideration. NP concentrations in the blood can still be detected even 24 h after a single administration. Therefore, sampling should be done beyond the 24 h mark (such as past 96 h or even 168 h) to ensure that sampling is sufficient to verify the full PK profile. The liver and spleen are also key compartments for phagocytes, and accumulation within these tissues should be measured during preclinical development.

### 2.2. Analytical Characterization to Evaluate the Plasma, Tissue, & Tumor Disposition of NPs

To understand why the disposition, safety, and efficacy can be so challenging to characterize, it is important to compare the analytical challenges of NPs to that of other drugs. However, disposition of NPs is not dependent on the cytotoxic payload, but the release of the drug payload, thus requiring a new nomenclature describing NP pharmacology. The “active” portion of the NP agent (i.e., the drug or other chemical substance to be delivered and provides mechanistic anti-tumor effects) has the same distribution profile as the carrier until it is released or unconjugated from their carrier [25,26,27]. When categorizing each part of the process, the term “encapsulated” or “bound” fraction was donned for the portion of drug that is inside the carrier (such as with liposome) or attached (such as a conjugate or dendrimer), respectively [25,26]. The term for the active drug that is outside or released from its carrier, and thus open to both perform mechanistic action as well as be affected by traditional ADME mechanisms, is the “released fraction” [25,26]. In this way, the encapsulated/bound form acts as a prodrug until the agent is released. In addition, the encapsulated drug will show differing PK from released drug, such as displaying a prolonged circulation in blood and potential accumulation within tissues. While such separations of encapsulated/conjugated and released forms are simple within the blood, the separation assays do not exist yet to provide an encapsulated/released fraction from tissue samples without using advanced and invasive techniques (e.g., microdialysis) [25,26]. Hence, we can measure the “total” faction, evaluating both forms of the drug, within these sample matrices. Additional discussion of the differences in the PK of conventional SM chemotherapy compared to nanocarriers has been reviewed, and recommend the review by Golombek et al. [28].

All forms of the drugs must be evaluated to appropriately assess how the disposition of NP drugs relate to their efficacy and toxicity. The most common analytical techniques utilize methods such as solid-phase separation (SPS), which have a long track history and have successfully been used to separate encapsulated liposomal agents from plasma samples [10,26]. Other NPs have utilized filtration or size exclusion to separate formulations where size is adequate to provide separation (such as dendrimers, polymer conjugates, or ADCs); but these methods have been problematic in the plasma due to recovery of protein-bound drug and non-specific binding to filtration devices. Finally, the agents which may require chemical conversion for activity (such as camptothecins), forceful conversion can be used to evaluate total and released concentration of plasma and tissue [29,30]. Additional concerns in the evaluation and calculation of tumor PK variables are discussed later in this chapter.

As mentioned earlier, the MPS is an important contributor to the overall disposition of NPs due to the variability in cellular function, resulting in the high, clinically-relevant inter-patient variability due to this non-traditional clearance pathway [31]. Upon entering the bloodstream from an IV infusion, plasma proteins and immune cells (including phagocytic cells like monocytes, neutrophils, and dendritic cells) will be the first entities to interact with NPs [32]. Although there is still much to learn about the interaction between NPs and immune cells, the uptakes of NPs by the MPS are at times engineered, purposely designed to stimulate the immune system, or results in unwanted side effects. Later in the chapter, discussion will focus on how the MPS plays a key role in the PK (concentration over time) and PD (efficacy and safety) of NPs.

## 3. Biological Concepts of Nanoparticle Delivery

### 3.1. Enhanced Permeability and Retention (EPR) Effect & Passive Targeting

Once a tumor begins to grow, its interaction with non-malignant cells leads to the development of the tumor microenvironment. New tumor vasculature is different than that of a healthy one, and leads to significant changes in the structure of the blood vessels surrounding the tumor, resulting in more porous vasculature within the tumor microenvironment [33]. Additionally, the lymphatic drainage system is commonly affected, causing an accumulation of intra-tumoral contents [34,35,36]. This process is called the Enhanced Permeability and Retention (EPR) effect and it can be exploited to enhance drug delivery of NPs. The EPR effect results in a passive targeting of NPs, where NPs accumulate in the tumor tissues by passing through the leaky tumor vasculature [34,35,36]. If the diameter of the NP is less than the vasculature pore, the NP can diffuse through that leaky vasculature pore and can linger in the interstitial spaces for an extended period [37,38]. This allows for an increased amount of drug to reside within the tumor, allowing for potential increase cytotoxic payload release, ultimately increasing the concentration of drug that can have a direct anti-tumor effect.

The size of the NP determines whether it will undergo passive targeting. Proteins such as transferrin (90 kDa) and IgG (160 kDa) were able to accumulate in the neoplastic tissues, while smaller proteins (e.g., neocarzinostatin [12 kDa], ovomucoid [29 kDa]) did not [35,36,39]. NPs that are too small will undergo passive targeting and thus will not lead to accumulation in the neoplastic tissues. In a study by Maeda et al., 40 kDa (~4.8 nm in diameter) was determined to be the cutoff in size for molecules that were able to go through the process of passive targeting [40]. On the other end of the range the EPR effect can still be seen with particles that are 10^4^ kDa, but the speed and efficiency of the intracellular uptake may be reduced [40]. Additionally, how well the EPR effect can be used to treat tumors depends on the concentration of each NP in the blood. Each type of tumor has different pharmacokinetics and distribution so not every tumor has the same EPR effect. Some tumors may allow for more efficient passive targeting for a specific NP, but types of cancers may not. Also, the release rate of the drug is an important factor that needs to be taken into consideration with the EPR effect. A study reported that NPs that release their contents at a slower rate achieve higher concentrations in tumor tissues than NPs that release at much faster rates [39]. This relationship shows that due to the accumulation, the NP will linger around in the tumor tissue longer and will have a better chance of releasing its contents at the correct location. The EPR effect and passive targeting are only one example of the factors involved in drug delivery of NPs. There are still many more than can increase or decrease the concentration of drug.

Overall, the EPR effect has served as the primary basis for the delivery of nanocarriers into tumors within clinical practice. However, the apparent heterogeneity in the EPR effect within different tumor types, coupled by the difficulty in objectively measuring this effect, makes it difficult to apply in clinical evaluations and patient care. Thus, limitations and challenges in understanding the multiple factors that can influence the EPR effect must be addressed; some of these are further discussed in Section 4 and Section 5.

### 3.2. Mechanisms & Factors Involved in Nanoparticle Uptake, Distribution, and Clearance

When NPs first enter the bloodstream, there are two main biological entities that these NPs will interact: plasma protein (mainly opsonins) and circulating immune cells. Opsonins consist of various protein substances that bind to the surface of foreign materials, including immunoglobin and complement factors. The process of opsonization, where opsonins attach to the surface of a NP, can have a significant effect on the overall diameter/hydrodynamic size and charge of an agent. Dynamic light scattering (DLS) is commonly employed to quantify the hydrodynamic size in solution at baseline during characterization of an individual agent, but coating the NP in the presence of plasma proteins can cause a ‘growth’ in the size of an agent. This is why some groups have come to report a “true” hydrodynamic size of an agent by incubating agents in serum before using DLA as this size will be more relevant in terms of the potential interactions within a biological system. To illustrate this point, a study evaluating gold colloids (~30 nm hydrodynamic size) showed a significant change in their diameters before and after opsonization in human serum (34.4 ± 0.2 nm and 94.8 ± 0.2 nm, respectively) [41,42]. Similar results were also observed for 150 nm gold colloids, with changes in hydrodynamic size from 149.8 ± 0.7 nm to 263.6 ± 4.7 nm before and after opsonization, respectively. As the surface of these colloids was altered, observable increases in the zeta potential were also observed in both the 30 nm colloids (−38.2 ± 1.2 mV and −16.4 ± 0.6 mV, respectively) and the 150 nm colloids (−46.3 ± 0.9 mV to −20.4 ± 1.9 mV, respectively) [41,42]. Based on these data, opsonization should be taken into consideration when characterizing NP formulations due to the profound effects it has on both size and charge.

The MPS cells (monocytes/macrophages) are the primary mechanism of NP elimination from circulation. In normal human physiology, these cells usually serve to remove offending pathogens from circulation and migrate to inflammatory sites due to complement and cytokine signaling proteins [43,44]. The ingestion of these NPs occurs by endocytosis, which includes the internalization mechanisms of phagocytosis and pinocytosis. Phagocytosis can further be sub-divided based on the endocytic mechanism, including complement receptor-mediated, Fc-gamma receptor (FcyR)-mediated, and scavenger receptor (SR)-mediated mechanisms. Similarly, pinocytosis is sub-divided into macropinocytosis, clathrin-mediated, caveolin-mediated, and clathrin/caveolin-independent endocytic pathways. Because of the specificity required to activate specific pathways, different receptors are necessary to recognize NPs based on their physical and chemical properties [42,45,46,47].

Phagocytosis relies on FcɣRs or complements receptors that require the opsonization of NPs by IgGs and iC3b, respectively, in order to bind the appropriate surface receptors and initiate phagocytosis. On the other hand, scavenger receptors are variably expressed on a variety of cells (including dendritic cells and macrophages of the MPS) function on molecular pattern recognition and are opsonin-independent. It is important to note that several classes of scavenger receptor exist, including classes A to F. The most well-known scavenger receptors on macrophages are Class A (e.g., CD204, CD162, MARCO), which binds to a broad array of ligands, including: proteins, lipoproteins, and pathogen-associated molecular patterns (PAMPs) on pathogenic Gram-positive and -negative bacteria [48,49,50,51]. Another type of scavenger receptor is Class B receptors (such as CD36, primarily serve to bind low-density lipoproteins (LDL), which oxidize lipoproteins and phospholipids. An example of a Class C receptor is CD206 (a mannose receptor found on macrophages) which binds to carbohydrate moieties using a calcium-dependent lectin receptor. Other scavenger receptors identified as Class D, E or F receptors bind a variety of ligands.

Receptor-mediated endocytosis is a complex mechanism performed by specialized cells that occurs using specific or non-specific receptors [52,53,54,55]. Chen et al. describes a theory of a non-specific opsonization on the surface of a NP then binds to receptors on the cell membrane of the patient’s immune cells, resulting in phagocytosis due to a lowering of membrane tension [56]. This action requires the spontaneous actin polymerization to occur in order to extend the cell membrane to create the phagocytic vesicle [44]. The size of these vesicles vary based on the size of the NP being engulfed but is always in excess of 250 nm. Another active process is receptor-mediated endocytosis, which involves the uptake of NPs via membrane surface receptors [57,58]. The difference between these two internalization methods (i.e., phagocytosis versus receptor-mediated endocytosis) is based on varying mechanisms to generate their vesicles (actin filaments for phagocytosis and clathrin-coated pits for receptor-mediated endocytosis). Alternatively, pinocytosis, a receptor-independent passive internalization pathway is fluid-phase, and typically results in the uptake of insoluble particulate, enzymes, immune complexes, and lipoproteins.

Phagocytic and pinocytic pathways require active transport that thus relies on actin polymerization to occur [42]. To evaluate this theory, cytochalasin D (an inhibitor of actin polymerization) was incubated with RAW264.7 murine macrophages and assessed the inhibitory effect on the internalization of gold colloid particles (30 nm and 150 nm) [42]. Overall, the presence of cytochalasin D resulted in reduced uptake of gold colloids, regardless of size or at lower doses of particles. On the other hand, at increasing concentrations of cytochalasin D, the uptake of the larger 150 nm colloid particles was decreased to a greater degree (~50%) compared to the smaller colloids. To differentiate if this difference in uptake was due to a phagocytic or pinocytic mechanism, 5-(N, N-dimethyl) amiloride hydrochloride (an inhibitor of Na+/H+ ATPase membrane pumps, and thus micropinocytosis) was added and incubated with cells. As the addition of the second inhibitor resulted in no change to the decreased uptake of either gold colloid, it can be concluded this internalization was due to a phagocytic pathway compared to a pinocytic one [42].

To evaluate the role of clathrin-mediated endocytosis, chlorpromazine was used to treat cells in vitro. Chlorpromazine is a well-characterized inhibitor of this pathway. The addition of chlorpromazine reduced the uptake of 30 nm colloids by RAW264.7 cells by ~50%. However, the effect on 150 nm colloids was negligible. A similar effect was seen when scavenger receptors were removed from the cell surface. This suggests that the smaller colloids could have a near-complete inhibition of cellular uptake if clathrin-mediated endocytosis and scavenger receptors were the only pathways of ingestion. However, when this theory was tested in RAW264.7 cells (which lack scavenger receptors) and incubated in the presence of chlorpromazine, a similar difference in the 30 nm gold colloid uptake was observed. This would suggest that scavenger receptors may not be involved in the clathrin-mediated phagocytic pathways [42]. As the inhibition of clathrin-mediated pinocytosis or scavenger receptor-mediated phagocytosis eliminated the uptake of gold particles, other endocytic pathways must also be involved. To test this pathway, filipin III, a specific inhibitor of caveolae formation, was used in gold colloid uptake evaluation. The degree of decrease was significant when compared to the negative control, suggesting that caveolin-mediated mechanisms are partially responsible for particle uptake [42].

The cells of the MPS, especially macrophages, express numerous receptors on the surface of their membranes to interact with their environment. Some of these receptors include complement receptors (CR), scavenger receptors (SR), fibronectin, receptors against IgG fragments (Fc), and glycoproteins to recognize receptors [59,60,61]. Similarly, receptors that respond to specific pattern recognition, such as SRs (e.g., CD36, CD204) and the mannose receptor (CD206), are primarily involved in receptor-mediated endocytosis [62,63]. Of particular interest, CD204 has been found to contribute to non-specific interaction (and thus non-specific uptake) of NPs which contain surface-functionalized materials (e.g., antibodies, synthetic polymers) [64]. These receptors on macrophages are also known to contribute to liposomal endocytosis [65,66]. Most of the surface receptors on macrophages are expressed based on the function of the phagocytes, tissue/species origin, and level of activation. Liu et al. studied varying clearance rates of liposomes from blood in mice, rats, and humans [67,68]. Within mice, liposomal uptake was mainly mediated due to direct interaction with macrophage receptors in the liver. However, rats demonstrated a stronger affinity for opsonin-dependent liposomal clearance within the liver. Human sera exhibited a higher activity of serum opsonins, though this was suggested to be due to C3 of the human complement system, highlighting the important role the liver plays in liposome uptake and its connection to the complement system [67,68].

The complement system consist of > 30 membrane-expressed receptors and circulating proteins and has been show to play an essential role in the immune response [69,70,71]. This system is further broken down into three activation pathways: (1) classical, (2) lectin, and (3) alternative pathways. Each pathway requires a distinct order and recognitions of components and events to cause pathway initiation—but the later stages of all three pathways utilize the same compounds. After successful activation of the complement pathway, the primary resulting product (opsonins [C3b/iC3b], the membrane attack complex [MAC], or anaphylatoxins [C3a, C5a]) exert a protective effect via opsonization, lysis of invading cells, or activation of surrounding immune cells, respectively [71]. This opsonization is essential for phagocytosis to occur efficiently by activated immune cells to ingest the invading particulate. However, it is also known that activation of the complement system can result in complement activation-related pseudoallergy (CARPA), a hypersensitivity reaction due to anaphylatoxin after IV administration of liposomal agents, such as Doxil [72,73,74]. Therefore, the complement system, and factors that can influence its activation, can greatly impact a NP’s distribution and tolerability.

It is clear that the various methods of cellular internalization of NPs are governed by several stimulatory and inhibitory signals/receptors—the recognition by which need to be considered to produce the best response and safety profile. How these pathways and regulators of internalization work in concert between tumor and professional phagocytic immune cells to modulate NP disposition remains to be fully elucidated. This effort has been hindered due to the complex and changing nature of these cells between cancer types and depending on the stage of tumor development [75,76]. From a clinical perspective, how the evaluation of these pathways can be incorporated into the current NP dosing paradigm also needs to be further evaluated to improve outcomes.

### 3.3. Is the EPR Effect Real? Active Pathways as the Primary Mechanism of Nanoparticle Uptake

Historical dogma would suggest that the primary route of NP entry into tumors is a passive process, the NP entering through gaps (found to be up to 2000 nm) between endothelial cells in the tumor vasculature [77,78]. However, recent evidence has been shown that supports that more active processes (i.e., trans-endothelial processes) are responsible for the uptake of NPs into tumors [79]. A recent study by Sindhwani et al. evaluated the role of alternative processes to passive diffusion using U87-MG, 4T1, PDX, and MMTV-PyMT mouse models either before or after whole mouse fixation [79]. This would, in theory, mean that NPs circulated after fixation could only enter the tumor via passive transport (due to existing gaps between cells), whereas mice undergoing fixation after NP administration could have NPs enter the tumor space by either passive or active mechanisms. Small molecule cisplatin and three different sizes of gold NPs (15 nm, 50 nm, 100 nm) were administered within these models before tumors were resected and analyzed by ICP-MS, 3D microscopy, and TEM. After circulating 50 nm NPs for 4 h, tumor concentrations between pre-fixed and post-fixation models were significantly different (0.10% ID/g versus 2.01% ID/g, respectively) [79]. Microscopy confirmed that trans-endothelial processes did not contribute to NP distribution within the pre-fixed mouse models. Furthermore, 49% of gold NPs extravasated in post-fixation animals (compared to 0% in pre-fixed animals), suggesting the dominant pathway of NP entry into tumors is using trans-endothelial pathways [79]. However, the addition of general or specific inhibitors of trans-endothelial pathways were not evaluated. While additional studies are necessary to clarify these results and determine which active mechanisms are predominating, this new information of NP uptake could lead to new strategies to enhance the delivery efficiency of NPs to tumors.

## 4. Tumor Microenvironment Factors that Affect Disposition of Nanoparticle Agents

**Figure 2:**
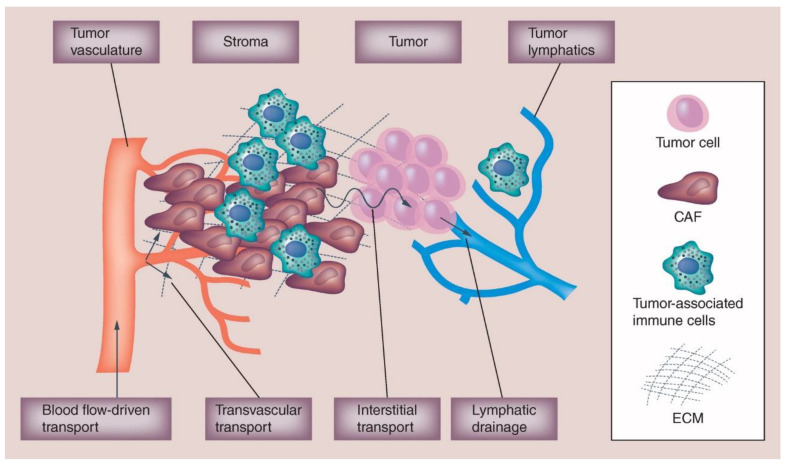
Complexity of the tumor microenvironment. The tumor microenvironment includes extracellular matrix (ECM), which poses multi-faceted barriers to drugs’ transport. The dense tumor stromal tissue, which is composed of collagens, fibronectin and hyaluronan, an abundance of cancer-associated fibroblasts, and aberrant interactions between infiltrating tumor-associated immune cells, cancer cells, and cancer-associated fibroblasts (CAF). Reproduced with permission from Lucas et al. [23]. Copyright Future Medicine Ltd., 2017.

One of the factors which complicate the treatment of solid tumors is the unique microenvironment that surrounds and forms the interior of each tumor. The tumor microenvironment (TME) is made up of the surrounding blood vessels, immune cells, cytokine messengers, soluble proteins, and the extracellular matrix (Figure 2) [80,81,82,83]. The tumor communicates with the microenvironment to activate angiogenesis and immunotolerance as well as control the growth of the tumor cells themselves. The interplay of these TME factors will influence the disposition of NP agents, making their PK and PD harder to predict.

### 4.1. Vascularity: Perfusion & Permeability

For NPs to have mechanistic anti-tumor effects, the active drug and the carrier must first move into the tumor from the systemic circulation for NP to diffuse to tumor interstitium. As mentioned, tumor vasculature is more permeable than normal tissues due to the larger pore size (100 to 780 nm vs. < 6 nm) [84,85,86,87]. NP extravasation into tumors is favorable in this ‘leaky’ environment, especially for NPs that are greater than 100 nm in diameter and allows for higher concentrations of drug in the tumor extracellular fluid [84,85,86]. However, the increased leakiness of the vasculature may also negatively affect NP delivery by way of increased interstitial fluid pressure in the tumor and blood flow stasis due to its high variability [86,88,89]. To take advantage of the leaky vasculature of tumors, several studies have attempted to make the vasculature more ‘normalized’ (i.e., structure and function more similar to normal tissues) through the exposure of pro- and anti-angiogenic signals [86,88]. The goal of these normalizations is to affect tumor permeability, which is typically less in normalized vasculature than in abnormal vasculature; but normalized tumor vasculature is still significantly more permeable than in normal tissues [88,90]. Several types of anti-angiogenic agents are currently in use within the clinic (e.g., bevacizumab, sorafenib) and commonly utilized in combination with chemotherapeutics. While higher doses of these agents result in anti-tumor effects by depriving tumors of necessary nutrients to grow (and limiting NP tumor delivery), lower doses have been attempted to achieve ‘normalized’ vasculature. However, finding the correct level of VEGF inhibition has evaded clinicians as the vasculature needs to be ‘normalized’ while still being able to be perfused. Furthermore, the effects of normalized vasculature appear to only be achieved for 2–5 days, but due to antiangiogenic therapies, cellular apoptosis leads to vascular regression which limits the use of NPs [86,88].

Once in systemic circulation, NP pharmacokinetics are influenced by the hydrodynamic forces and fluid-formulation interactions that accompany the body’s blood flow. NP experiences tumbling/rolling dynamics as well as particle-cell interactions while in circulation, and these forces influences the potential of NP reaching its final target destination. Therefore, the interaction of the NP with the endothelial walls, through particle–cell and receptor-ligand interactions, as it travels through fenestrated tumor blood vessels is a very important design consideration and highly relevant to drug delivery [89].

The physiological factors of the tumor vasculature have been found to lead to decreased NP delivery to tumors. These factors include heterogeneous blood supply, uneven permeability, and larger transport distances in the interstitium [6,32]. Tumor periphery has a high perfusion rate, but it is not as permeable as the necrotic core. The tumor periphery has been shown to have decreased permeability and increased blood volume making it more difficult for NPs to move into the tumor core [91,92]. This has been shown through the use of human adenocarcinoma xenograft models [87,92]. Window chambers were used to observe labeled liposomes, and investigators found a significant accumulation in xenografts compared to normal tissues. Also, the heterogeneous distribution in the tumor showed increased accumulation in the peripheral vasculature [87,92]. Similar results were also seen when 111-In labeled micelles was studied [93,94,95]. These studies show that NPs do not have problems crossing vascular barriers, but with perfusion and navigating once within the tumor microenvironment.

The role of heterogeneity of the tumor vascularity of triple-negative breast cancer (TNBC) was evaluated in a study using genetically engineered mouse models (GEMMs) to measure efficacy and drug delivery. This study compared a NP (PEGylated liposomal doxorubicin, PLD), to small-molecule (SM) formulation of doxorubicin [27]. Two GEMMs with differences in TNBC subtype were utilized in this evaluation: C3-TAg (basal-like) and T11 (claudin low) TNBC subtypes [27]. Plasma AUC was similar between the NL-doxo and PLD in both tumor models, but the AUC for PLD was 2-fold greater in the C3-TAg model than the T11 (*p* < 0.05) [27]. Using immunohistochemistry (IHC), each tumor model was evaluated for the total amount of macrophages, vascularity, and collagen after administration of PLD (6 mg/kg IV x 1) for both the NP and SM. There was greater efficacy with the PLD in C3-TAg than with T11 [27]. After dosing the T11 tumors with PLD, the microvessel density (MVD) decrease by 30% from baseline (*p* < 0.05) [27]. The T11 tumors also had higher levels of VEGF-a compared to the C3-TAg tumors (*p* = 0.003) and PLD had more of an effect on VEGF-a levels than SM doxorubicin [27]. The results suggest that tumor-specific differences in the microenvironment, such as MVD, impacts NP delivery, but not SM formulations. This study illustrates the variability in delivery and efficacy when using NPs for treatment. The tumor microenvironment is different between tumor models and this can impact the PK of NPs used. This study also shows that not only does the tumor environment affect the delivery of NPs, but the administration of NP can lead to changes in the tumor microenvironment and hinders its function. NPs used for one type of tumor (e.g., TNBC models) may not be able to be used in the others due to different TME factors can impact rates of clearance and distribution. The similar tumor associated macrophage (TAM), MVD and collagen at baseline in GEMMs suggest alternative tumor factors (e.g., pericytes or tumor perfusion) may affect the tumor delivery of NPs but not SMs. Also, changes in vascularity and VEGF-a overtime may reduce tumor delivery of PLD in T11 tumors.

### 4.2. Stroma

The majority of a tumor is comprised of the basement membrane, fibroblasts, and extracellular matrix proteins (e.g., collagen, fibrinogen); collectively, termed the ‘stroma’ [96,97,98,99]. These facets of the tumor stroma then interact with tumor cells through inflammation and matrix building activity (ex. unregulated fibroblasts lead to continuous proliferation). NP distribution by diffusion and convective transport has been classically negatively affected by the tumor stroma due to the excess extracellular matrix, an effect not as prominent with small molecule agents [100,101,102]. For instance, collagen in the stroma also can decrease the ability of NPs to get to their target [81,103]. Intra-tumoral transport of several NP agents, including PLD, DaunoXome, and Abraxane, has shown to decrease within tumors and correlated with dense collagen fibers [104,105]. Additionally, the rate of growth for tumor cells decreases the further the cells are away from the blood vessels because it needs a constant influx of nutrients to replicate [106,107,108]. Because most chemotherapy agents, and some NPs, work best on cells with rapid turnover rates, problems with resistance begin to arise with tumors that are farther away from the vasculature because these tumor cells proliferate at a slower rate [106,107,108].

One option investigators have researched for dealing with the troubles caused by the stroma is to target inhibiting factors, such as fibroblasts, within the stroma by using NPs [109,110,111]. This method focuses on decreasing the mass associated with fibroblasts so that there would be greater perfusion for drug delivery [110,111,112,113]. One such study used a docetaxel NP conjugate composed of PEGylated and acetylated carboxymethylcellulose to target fibroblasts in an orthotopic murine breast cancer model [114]. Fibroblasts internalized over 85% of the tumor-associated NPs, resulting in a near-complete cellular fibroblast death, leading to a significant increase in tumor perfusion and reduced IFP within a week after administration [114].

Another method of getting over the stomal barrier would be to use NPs to re-engineer these rate-limiting factors into positive therapeutic tools. An example of this is the use of lipid-coated DNA protamine complexes loaded with plasmids to target tumor fibroblasts, re-focusing their efforts to generate cytotoxic secreted proteins produced by these cells [115]. Normally the off-target delivery of NPs to fibroblasts is not ideal for the treatment of desmoplastic tumors. However, by loading plasmids encoding sTRAIL (a secretable TNF-related factor) into lipid-coated protamine DNA complexes to target these cells, these fibroblasts produce sTRAIL at clinically-relevant levels [115]. In a desmoplastic murine model of bladder carcinoma, after only three doses of sTRAIL-loaded complexes, 70% of fibroblasts were producing the new sTRAIL protein after three doses, causing apoptosis in nearby tumor cells [115]. These results were also confirmed within human pancreatic cancer models, a traditionally difficult to treat cancer using small molecule chemotherapy.

So far, there are no clinically approved (i.e., included in evidence-based guideline recommednations) combinations or strategies to modulate the tumor stroma to enhance nanoparticle delivery and/or safety.

### 4.3. Interstitial Fluid Pressure

Interstitial fluid pressure (IFP) is a factor contributing to the convective flow of nutrients, and drugs and in combination with the EPR effect (i.e., increased permeability of the tumor vasculature), contributes to the variable disposition of NPs. In the absence of a tumor, the convective flow is low and fluid is taken into the interstitial space, lymphatic ducts through the net negative pressure between blood vessels and the interstitial space [116]. NPs are efficient in their transportation within tumors when the IFP is low, yet due to various factors of the tumor microenvironment, the IFP is commonly increased within tumors [116,117]. Methods for de-bulking tumor stroma have thus been studied to decrease the IFP because when it is elevated, the pressure will limit extravasation, making it difficult for NPs to penetrate tumors and areas further away from blood vessels [118,119]. Such methods studied include using losartan to de-bulk the tumor stroma through its ability to inhibit the angiotensin system [120]. This approach would assist in restoring vessel profusion in the tumor microenvironment and thus decrease the IFP due to the size of the tumor.

Combined interactions between tumor blood flow and intra-tissue pressures ultimately leads to an increased IFP and a decrease in the blood flow around the tumor. To assess the effect of this relationship on NP disposition, a study was done to measure concentrations of NPs in a murine metastatic breast cancer tumor model (MDA-MB-231). This study measured IFP by using a modified wick-in-needle technique and tumor perfusion, and NP distribution was imaged using iohexol (a CT contrast agent) to view intra-tumoral liposome accumulation. The results of their study showed a strong correlation between IFP and tumor perfusion (Spearman’s r = −0.88 to −0.97, *p* < 0.0001) [119]. There was also a significant correlation between IFP and NP accumulation (Spearman’s r = −0.64, *p* = 0.0029), but this correlation was weaker and more dependent on whether a subcutaneous model or orthotopic model was used (Spearman’s r = −0.64, *p* = 0.0029) [119]. In conclusion, the combination of an increased IFP and decreased tumor perfusion demonstrates as another barrier to effective NP distribution in tumors.

### 4.4. Mononuclear Phagocyte System

The MPS is part of the innate immune system (IIS) and is one of the biggest contributors to the PK and PD disposition and variability of NPs in both animals and patients. Most NPs are created to extend the time they spend circulating and made to avoid mechanisms that quickly clear SM drugs from the body. The most common uptake pathway for NPs is usually opsonin adhering to the surface of NP, then signals phagocytosis of the NP, affecting the concentration of drugs in tumors and tissues [121]. This phagocytic mechanism is a complex process that is not well understood in the context of drug uptake and how the MPS affects NP clearance from the blood and distribution into tissues or tumors that may occur in a completely different manner. To better understand the two mechanisms of NP delivery within the tissue, the terms ‘capture’ and ‘hijack’ were created [122]. In the ‘capture’ model, NP uptake occurs after the NP moves from the blood to the area of interest (e.g., liver, tumor, spleen) [122]. However, in the ‘hijack’ model, a NP in the systemic circulation is phagocytosed into the MPS cell, where the cell then travels into the site of action [122]. Both the capture and hijack models affect the PK of NPs, but the influence, occurrence, and prevalence of these methods of NP delivery are not well studied.

Animal models for SM drugs do a sufficient job of predicting the PK of these drugs in humans; but for NPs, this process is more difficult because of the MPS effects on NP clearance, efficacy, and target site delivery. When measuring the number of macrophages in matching flank and orthotopic xenograft cancer models (consisting of breast, ovarian, endometrial cancers, and melanoma), the MPS presence was significantly different between not just tumor types, but also among tumor cell lines of the same tumor type as well as the implantation site of the tumor (i.e., flank versus orthotopic) [123]. The results show the importance of knowing the variation in MPS presence within each preclinical model and the extent of orthotopic implantation when selecting an appropriate animal model.

In a study examining GEMM of TNBCs, there was a positive association between variation in tumor microenvironment features and efficacy of PLD [27]. These differences in drug disposition were, however, not seen with SM drugs. The results of this study support examining the tumor microenvironment and tumor type before administering NPs to patients to ensure efficient delivery and maximum efficacy. Administering a NP in an environment that has decreased extravasation or to a resistant tumor could lead to treatment failure. There are still many questions as to why two tumors with similar genetic makeup respond differently to NP therapy. There is a need for information regarding which factors alter tumor delivery of NPs. Studies in both humans and tumor models need to be conducted to better predict which tumors will respond best to NPs.

Up to 60% of a tumor can be made up of macrophages, and these are commonly referred to as TAMs [124,125]. These TAMs play a variety of roles associated with tumors, including the delivery of NPs to the tumor matrix as well as help with immune suppression and metastasis [126,127,128]. Metabolites of NPs, such as radiolabeled paclitaxel poliglumex, were found in TAMs at levels 100 to 1000 times greater than that of the tumor cells [129]. In a separate study, the polymer formulation of paclitaxel poliglumex was attached to gadolinium (an MRI contrast agent), where it was found to be phagocytosed by TAMs and located throughout the tumor [130]. Within the tumors, TAMs/NPs are mostly found in the area of necrotic cells [129,130]. Similar results were also seen when evaluating the effect of TAMs on the delivery of PLD and SM doxorubicin in C3-TAg and T11 tumors. At baseline, most TAMs were determined to be in the necrotic core and tumor capsule in both tumor types [27]. There was very little consistency between the two tumor models for the SM doxorubicin, but for the PLD there was a drop in TAMs at 24 h [27]. The T11 tumor had a significant decrease in TAM infiltration into the viable tumor area compared to the C3-TAg (37.2% vs. 6.6%, *p* > 0.05) [27]. These results show that PLD may be cytotoxic to TAMs initially, leading to a decrease in the first few hours of therapy. In addition, TAMs uptake NPs and either clear them from the body or help deliver them into the tumor; so any disruption in the number of TAMs can lead to significant PK consequences.

To examine the impact that this MPS effect has on PK and PD disposition, as well as efficacy of NPs, SKOV-3 ovarian cancer, and HEC1A endometrial cancer orthotopic xerographs were treated with both PLD and SM-doxorubicin [123]. These two preclinical cancer models were chosen because SM-doxorubicin is more efficacious in endometrial cancer than PLD. The two models had similar concentrations of doxorubicin in the plasma, liver, and spleen, but the ratio of tumor to plasma AUC_0–96h_ of PLD was much higher in the SKOV-3 model than in the HEC1A model leading to a better survival benefit when using PLD in the SKOV-3 model [123]. These results suggest that NP-based therapies need to account for heterogeneity in the tumor microenvironment, especially factors relating to and/or modulating the MPS, within preclinical models.

While there are no clinically approved MPS-associated markers for altering NP therapy, these cells have been a focal point in both precision dosing and theragnostic dosing strategies, evidenced by the analysis of these cells both as part of correlative analyses in several early-stage clinical trials of NPs and their extensive analysis in the preclinical evaluations of efficacy and toxicity.

## 5. Considerations in the Safety & Efficacy of Nanoparticle Agents

Although efficacy (i.e., anti-tumor effects) is the primary goal for the use of NPs in the treatment of cancers, safety is also a major component and motivator to the approval for human use. Much like any other cancer agent, NPs carrying cytotoxic payloads are going to be toxic to healthy cells even though they are meant to provide targeting towards tumor cells. NPs can cause these toxicities by several means, such as through the delivery/release of the payload release resulting in toxic drugs being delivered to healthy tissue. Ideally, encapsulated/conjugated NPs will circulate and accumulate within the tumor (such as due to the EPR effect) and release their cytotoxic payload within the intra-tumor extravascular space. However, when these NPs release their chemical payloads prematurely and away from tumor cells, the cytotoxic agents will commonly result in unwanted toxicities. Additionally, NPs can affect the disposition of other drugs causing an indirect drug interaction that leads to toxicity as well. Because safety is a priority, it is important that the preclinical models used accurately reflect human physiology and allometric scaling techniques use the most appropriate model. By better understanding, the toxicities present with NPs more informed decisions can be made when designing first in human studies.

### 5.1. Alteration of Pharmacodynamic Toxicity in Nanoparticles

In addition to altering the pharmacokinetics of small molecule drugs, NPs were developed to improve the safety profile of these cytotoxic agents. A major problem in cancer treatment is the inability of patients to tolerate their chemotherapy regimens, resulting in worsening outcomes due to lack of therapy and drug exposure. NPs are in theory more specific in nature and are designed to deliver the payload directly to the site of the tumor. The most studied example of clinically approved NPs being safer than SM agents is with PLD and doxorubicin. A meta-analysis conducted by Rafiyath et al. examined the differences in safety and toxicity between liposomal doxorubicin and conventional anthracyclines [131]. This group examined 2220 patients (1112 liposomal and 1108 conventional) and saw statistically significant decreases in the incidence of congestive heart failure, alopecia, neutropenia, and thrombocytopenia [131]. By using an encapsulation carrier to assist toxic drugs in reaching their site of action, systemic side effects are limited, and patients will be able to continue taking their drugs for a longer period of time.

### 5.2. Payload Release versus Nanoparticle Delivery to Target Organs

The strength of NPs and the reason they have the potential to be very effective in treating solid tumor cancers is that the carrier is able to specifically release its payload into the tumor. As stated throughout this chapter, this form of delivery is variable, and the payload does not always reach its intended target. Occasionally, there is a rapid release of drugs from the NP into the blood. This premature release will not be able to affect the tumor and will result in numerous side effects for the patient. When this phenomenon occurs, patients usually experience common chemotherapy side effects such as nausea, peripheral neuropathy, weakness and fatigue [132]. If NPs consistently release their payload early, patients will experience more toxicity (in line with systemic toxicities of the SM payload) than beneficial treatment.

In a study by Lai et al., self-assembling micelle formulations of dextran-doxorubicin (Dex-DOX) were measured in B16F10 melanoma–bearing Balb/c mice [133,134]. Samples with high molecular weight dextran (500 kDa) had higher release rates of de-conjugation, resulting in reduced doxorubicin drug levels reaching the tumor site when compared to lower molecular weight dextran (40 kDa) [134]. Due to the risk of a rapid release of drug from NPs into the blood, it is imperative that the analytical methods for measuring drug are specific. It is important to measure both the encapsulated/conjugated and released fractions for any NP agent to determine payload release kinetics. If the released fraction of the drug is not being measured in the tumor, but in other areas such as the liver or spleen, then it can be inferred that either a) the release is happening too soon or b) the NP is being cleared due to MPS-related clearance mechanisms—ultimately resulting in the active component of the drug carrier to be unable to reach its desired destination.

While it is imperative that the cytotoxic payload of a NP not release too early from its carrier, it is also crucial that the drug still releases at some point after being injected into the body. NPs that use encapsulation or conjugation to transport the payload to the active site must release the drug if there is to be any kind of effect. If there is no release of cytotoxic drugs at the active site, it would be as if there was no treatment was administered, even if significant NP accumulation occurs within the target tumor. One example of inefficient drug release is with SPI-077, a liposomal cisplatin formulation that had demonstrated promising results during preclinical studies conducted in murine lung cancer models [135]. In the preclinical studies, the total (encapsulated + released) tumor exposure for SPI-077 was significantly increased compared to small molecule cisplatin [135]. However, when SPI-077 was translated into the clinic, efficacy was low; ultimately resulting in the trial to be discontinued early [136,137,138]. Additional PK analysis showed a lack of platinum in the plasma and tumor extracellular fluid, and low platinum-DNA adducts within the tumor [139,140]. These results showed that the active cisplatin was not releasing from its liposomal carrier, limiting its anti-cancer effects. This study serves as the primary example to stress the importance of optimizing the release kinetics and making sure that both the encapsulated and released forms of NPs are properly measured.

### 5.3. Drug-Drug Interactions

Although NPs have a unique mechanism of action and mainly interacting within the tumor microenvironment, drug-drug interactions (DDIs) are still present and still need to be considered. Direct, classic DDIs are rare with NPs because the drug is protected by its carrier. It is not until the drug is released that the drug may interact with another substance. NPs are mostly affected by the tumor microenvironment, but indirect DDIs have been observed in the NP class. However, these indirect interactions are being used to improve NP efficacy, such as by increasing tumor delivery or improving the release of drug from the carrier.

One way to increase the NP penetration into tumors is to pretreat the tumor of interest [141,142,143]. In a study by Jain et al., the group examined how the presence of collagenase affected the penetration of antibodies and viral NPs [141,142,143]. The results showed a 2- to 3-fold increase in tumor penetration in collagenase pretreated tumors than tumors that had not received pre-treatment [141,142,143]. Similarly, tumors pre-treated with relaxin also have a 2- to 3-fold increase in tumor penetration due to its effect of degrading and structurally changing collagen [141,142,143]. Additionally, reduction of stromal collagen and hyaluronan from the use of losartan lead to increased vascular perfusion and thus a 74% increase of 5-FU tumor AUC compared to 5-FU use alone [144]. Another potential method to increase tumor delivery was to prime tumors with a low dose IV traditional cytotoxic agent (such as paclitaxel) and then administer the liposomal formulation. This method led to a two-fold increase in the delivery of the agent, but this delivery stems from the toxicity of the traditional agents which may also disrupt the MPS effect increase tumor delivery due to decreased systemic clearance and lack of optimized delivery. Conventional radiation has also been used with NPs, but the increase in drug delivery was small at 0.2- to 3-fold [145]. Hyperthermia is also a strategy that has led to an increase in intravascular drug release due to the use of thermally sensitive liposomes [146,147]. This process, however, can only be used in locations that can effectively conduct this therapy, limiting its use [146,147]. The administration of concomitant therapies all leads to increased risk for toxicities and with an increase in only moderate, better methods are needed to increase delivery.

On the other hand, studies have also looked at administering the NP first, then adding a NP modifying agent to improve drug delivery, instead of pretreating tumor using SM therapy before a NP is administered. One such example examined how adding Pluronic P85 could improve the delivery of PLD through blocking copolymers once the liposomal drug has accumulated in the tumor [148]. Pluronic P85 was injected at 1, 48, and 96 h after PLD administration in A2780 ovarian xenografts [148]. The release of doxorubicin from its carrier as well as delivery to in vitro tumor cells were both significantly increased [148]. This example shows that there is a potential to improve NP release with the additional administration of specific small molecule polymers. This could be an interesting strategy to better control and understand the PK of NPs.

### 5.4. Preclinical Model Selection: Variability in Patients & Animals

Selecting the correct preclinical model is imperative to obtaining accurate results that can be translated to the treatment of humans. The physiology of the preclinical animals that are used in research may not exactly emulate humans, so it is important to understand these differences in order to select the best model for testing.

The tumor microenvironment is one important factor that needs to be taken into consideration when determining which preclinical model most accurately emulates the human condition. Due to the numerous cell line models available for purchase and methods of implanting tumors (i.e., subcutaneous versus orthotopic implantation), it is reasonable to assume that variations in methodology could impact the tumor microenvironment. In a study by Lucas et al., the tumor microenvironments (specifically, macrophage presence) of several commonly used tumor models of melanoma, ovarian, endometrial, and breast cancers were evaluated [123]. Overall, significant differences existed in the macrophage presence within each tumor model, even between models of the same type of cancer. In addition, macrophage presence is different between subcutaneous versus orthotopic implantation of the same model. Due to the physiology of the tumor placement, orthotopic tumor models may be a better choice when trying to simulate a real patient, and the differences in tumor location could influence the microenvironment of the tumor causing a change in NP PK. Due to the known importance of the MPS in affecting NP disposition [25,124,149,150], these data suggest the importance of orthotopic implantation when possible and that preclinical models need to be chosen based on microenvironments that are similar to humans.

Another difference between the tumor microenvironment between humans and animal models is due to full or partial presence of the immune system. Many times, immune-deficient mice are used in pre-clinical studies to assess the drug’s direct effect on tumors and to aid in the growth of xenografts. With the lack of T or B cells in these mice, we cannot examine inhibitory or stimulatory relationships between NPs and immune system function, including combinations of nanotherapy with novel immunotherapies. For example, novel interleukin-12 (IL-12)-based nanotherapy is being researched because of its stimulatory effects of both NK cells and cytotoxic T lymphocytes [151]. By looking at the effects of NPs on the immune function in the tumor microenvironment, we can begin to understand how these therapies will behave in humans.

In a review article by La-Beck et al., the group discussed interactions between the immune system and the tumor microenvironment [149]. The tumor microenvironment is normally filled with cells that suppress antitumor responses (including T cells, TAMs, and MDSC) [150,152]. Certain TAMs (“M1-like”; pro-inflammatory) are crucial in the signaling that results in activating the immune system to fight against the tumors; whereas “M2-like” (anti-inflammatory) TAMs aid in tumor growth and metastasis [150,152]. In an immunocompetent TC-1 tumor model in vivo study, treatment with alendronate-loaded liposomes led to an increase in TAMs with an M2 phenotype [153]. This was compared with mice treated with vehicle drugs which exhibited an accumulation of M1-related TAMs in the tumor microenvironment [153]. Adding to this evidence, TGF-beta, associated with the cytokine profile of M2 TAMs, was increased when cultured macrophages phagocytosed liposomes [154,155]. These studies together show that an immune response induced by NPs may be contributing to the lack of improvement in efficacy, one that outweighs the cytotoxic payload they carry. Additionally, it is important that the animal models we use accurately reflect the human tumor microenvironment as much as possible. Ideally, we want the models used to represent the full human disease. If there are interactions with the immune system, these need to be noted and researched to better understand the effects induced by the administration of NPs.

**Figure 3:**
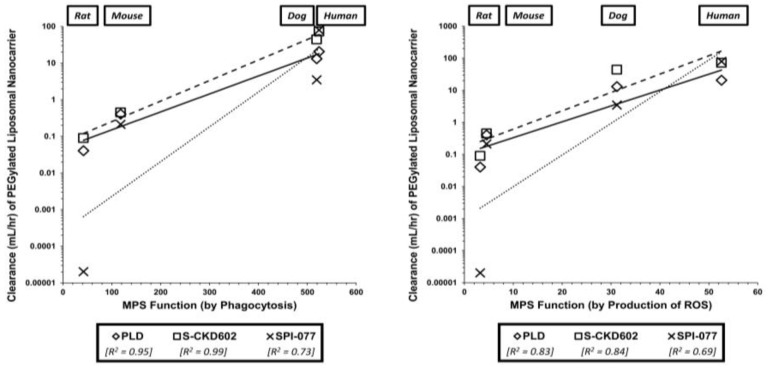
Activity of the MPS (phagocytosis and production of reactive oxygen species [ROS]) in monocytes from blood compared to the clearance of PEGylated liposomal nanocarriers in mice, rats, dogs, and patients. The ability to more accurately convert preclinical data into human patients may best be performed by measuring the factors responsible for NP uptake and clearance, such as the cellular function of the MPS. The mean values for three species are represented by individual symbols, with ◊ as PLD, □ as S-CKD602, and X as SPI-077. The exponential line of best fit for each group is represented by the lines. Overall, a positive association can be seen between cellular function and clearance of PEGylated liposomal nanocarrier agents. *Reproduced with permission from Caron* et al. *J Pharmacol Exp Ther. 2013, 347, 599–606.*

Another way to understand the PK and PD of NP drugs is to understand how their safety compares to SM drugs. The safety and distribution of SM drugs are better known and are relatively easier to predict than NP agents. In a study by Lucas et al., a unique ex vivo profiling platform of the MPS was used to compare the pharmacokinetic differences between multiple NP formulations of anthracyclines, including: PEGylated liposomes (i.e., Doxil), non-PEGylated liposomes (i.e., DaunoXome), micellar doxorubicin (i.e., SP1049C) and traditional small molecule doxorubicin (i.e., Adriamycin). These agents were then screened within common nonclinical models, such as SCID mice, Sprague-Dawley rats, and beagle dogs [156]. This MPS screening measured the function of MPS cells (via the amount of phagocytosis that was seen) for each given formulation [156]. MPS screening for mouse and rat blood emulated human MPS behaviors, demonstrating a trend in the reduction of phagocytosis of SM-doxorubicin > SP1049C > DaunoXome > Doxil [156]. This trend was most likely due to cytotoxic effects on monocytes and dendritic cells from the SM formulations and increasing protective properties of the various formulations from traditional systemic clearance mechanisms and recognition by the MPS [156]. Understanding this phagocytic activity can be crucial in developing new model species to predict the PK of NP agents. This is supported as baseline MPS activity (such as phagocytosis and ROS production) were previously associated with inter-species differences in the clearance of PLD and other liposomal NPs (Figure 3) [157]. If we are able to understand how much NP formulations are cleared by MPS cells, we can better predict how this will translate to humans. Additionally, only MPS profiling in rats could statistically differentiate between colloid forming drugs, which display aspects of nano-like disposition [156]. However, this information could still be useful as a potential method to screen for characteristics of colloid-forming drugs, so that we would be able to predict in vivo PK parameters.

Due to the MPS being an important factor in the clearance of the NPs, any decrease in its function could lead to safety concerns. This causes a significant risk for older patients who have immune systems do not function as well as compared to younger patients. In adults that are over the age of 80, there can be a loss of MPS function which would put them at risk for NP related toxicities [15]. This effect was examined in a Phase II trial with 60 elderly subjects who were treated with PLD (Doxil) for metastatic breast cancer [158,159]. The results of this study showed that as subjects got older, the PLD plasma half-life was extended (due to reduced clearance of liposomes by the MPS), which led to a higher incidence of Doxil’s most common side effect: palmar-plantar erythrodysesthesia (PPE) [158,159]. This relationship was especially true for subjects over the age of 80 years old [158,159]. Based on these results, older patients could be at risk of toxicity due to the impaired function of their monocytes. Without a functional MPS, the NP clearance is decreased and drug exposers for elderly patients would put them at a higher risk for toxicity.

MPS-related toxicity has also been seen with the use of gadolinium-based contrast agents. These agents are mainly used for magnetic resonance imaging (MRI) and come with very serious side effects of nephrogenic systemic fibrosis (NSF) [160]. NSF is characterized by thickening and darkening of large areas on the skin. Once contrast agents have been administered, gadolinium is engulfed by MPS cell mediators and transported to the three most common sites of MPS cell accumulation: the liver, spleen, and bone [161]. This could lead to toxicity, and an increased chance of patients developing NSF. Additionally, gadolinium stimulates macrophages to undergo iron recycling, which is likely the reason why iron accumulation in tissues is common in NSF patients [162,163]. Based on these two factors, there seems to be a trend that the MPS can be attributed to the mechanism of toxicity for gadolinium. Because of the way the MPS system can sequester foreign material, certain patients may be at more of a risk of toxicity, based on inter-patient differences in MPS function.

Understanding the effects of chemokines and other mediators in animal models is important to the disposition of NPs—but the end goal of human treatment is still something to be kept in mind. Chemokine effects should be taken into consideration when developing animal models, but direct comparisons to humans should be cautioned. For example, CCL5 is a more prominent chemokine in humans than CCL2, which is the reverse of many murine models.

The prominence of CCL2 in mice was shown in a study by Song et al., in which knock-out (KO) mice were used to isolate the effects of CCL2 and CCL5 on the PK of PLD [164]. This approach was used to see which cytokine had more of an effect on the PK of PLD. Compared to wild type mice, CCL5 KO mice that received PLD had higher encapsulated plasma AUC values (*p* = 0.05). CCL2 KO mice did not achieve a significant increase in encapsulated AUC, but there was a positive trend [164]. However, CCL2 KO mice did have a significant decrease of PLD accumulation in the liver and spleen (*p* = 0.038 and 0.014, respectively) compared to both CCL5 KO and wild type mice [164]. The results of this study suggest that CCL2 plays a more significant role in activating macrophages to and indirectly affecting PLD uptake and accumulation of PLD in MPS-related organs. Focusing on animal models that do not accurately represent the physiological state of humans may cause errors in targeting and evaluating disease states. It is important to have a great understanding of PK mechanisms for drug disposition before new therapies are tried in humans. When possible, chemokine activity should be matched to humans to be able to more accurately translate animal models.

### 5.5. Which Model is Most Appropriate for Allometric Scaling?

Almost all research is completed using animal models at some point in the development of a new therapy with the understanding that preclinical results can be successfully translated to humans. One way of doing this is to use allometric scaling, which involves using preclinical animal data to predict human PK parameters to determine a clinically relevant starting dose to use in drug development. While rats and dogs are two of the most commonly used animals in toxicokinetic studies of NPs based on their cost, their anatomy, physiology, and biochemistry are similar to humans—thus, size is the key difference between each species [165,166,167]. PK processes and body weight among mammals are compared using a power-log relationship to translate parameters like clearance from animal to human [168,169,170].

Allometric scaling techniques have not led to many benefits in translating the distribution of NPs from animal models to humans. For this reason, it is not heavily studied and there is a lack of literature in this area. In a study seeking to evaluate allometric scaling of PEGylated liposomal agents, the three most common models (mice, rats, dogs) were used to determine if non-standard physiologic variables provide more efficient PK parameter estimation in human [168]. While bodyweight is traditionally used in allometric scaling equations, Caron et al. demonstrated that MPS-associated variables (e.g., liver or spleen weight, liver or spleen blood flow, and monocyte count) also provide similar or improved linear associations [168]. The highest linear association to NP clearance was seen with Doxil, S-CKD602, and SPI-077 using total monocyte count (R^2^ values of 0.954, 0.989, and 0.933, respectively) [168]. Bodyweight also correlated well to clearance (R^2^ = 0.974, 0.977, 0.892) [168]. These results show the difficulty of using allometric scaling to determine dosing for humans and how a combination of physiologic factors (as compared to a single variable) may be necessary to improve the predictive quality of this technique. Having a good model is important to translational work because accurately predicting which components have the greatest effect on PK parameters can make scaling up to humans more of a realistic feat.

This stated variability and lack of effectively applying appropriate allometric scaling is compounded and has drastic effects when determining the starting dose of a first-in-human phase I clinical trial. Much of this difficulty comes from trying to explain the inter-patient PK variability between NP agents. In a meta-analysis by Schnell et al., the inter-patient variability of 9 liposomal and their matched non-liposomal (i.e., small molecule) agents were evaluated [171]. The coefficient of variance (CV%) of AUC as well as fold difference between AUC_max_ and AUC_min_ (i.e., AUC range) were used to assess PK variability within each trial [171]. Results of this meta-analysis showed that liposomal agents have much more variability between patients than SM drugs, with the CV% of AUC had a 2.7-fold (*p* < 0.001) difference for liposomal drugs compared to the SM formulations [171]. The trend remained similar for AUC ranges (16.7-fold differences, *p* = 0.13); however, results were not significant for this comparison [171]. This variability adds a significant cost to the development of NP agents as the PK variability results will lead to more time and resources needed in an individual study. For example, many more dose escalations were needed for NPs compared to SM drugs. This trend was highlighted in a meta-analysis by Caron et al., in which the group gathered information about the number of dose escalations and patients enrolled in phase I NP and SM trials [169]. The studies involving NP agents had a significantly greater number of dose levels than studies involving SM agents (7.3 vs 4.1, respectively; *p* = 0.008) [169]. With more dose levels, more resources are needed to account for an increased need for patients. At an average cost of ~$100–150,000 per patient in a phase I trial, the increased number of patients means a significantly higher cost to run these trials. The findings of this meta-analysis highlight the compounded effect of inefficient or non-translatable preclinical model selection and commonly applied allometric scaling techniques that can affect the overall cost and outcomes of NP studies compared to their SM counterparts.

### 5.6. Pharmacokinetic Parameters used to Describe Nanoparticle Tumor Disposition

**Figure 4:**
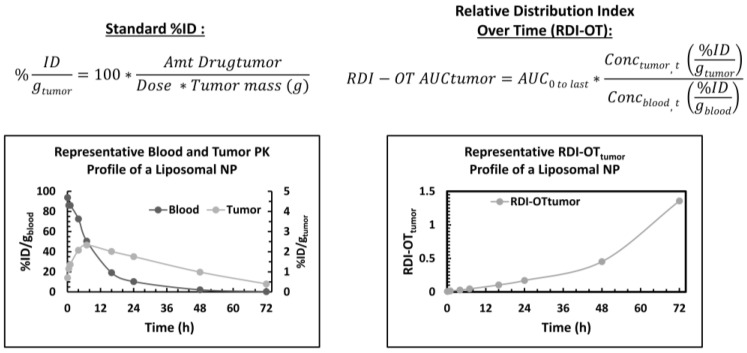
Calculating nanoparticle delivery efficiency using standard percent injected dose (%ID) calculation versus relative distribution index-over time (RDI-OT). The conventional calculation of tissue %ID represents the amount of drug in the target tissue at a single time point in time. However, RDI-OT is calculated for each time point within the profile, providing a new profile of the delivery efficiency of the nanoparticle over time compared to a single point in time.

Normally, PK parameters such as clearance and volume of distribution were used to describe the disposition of drugs, regardless of being a small molecule drug or a complex NP. These standard PK parameters work well for characterizing small molecule drugs, but there are concerns that traditional mathematical analyses may not provide enough information about tumor delivery due to the prolonged circulation affecting PK parameters, thus affecting the accuracy to describe NPs [170]. To combat this problem, Madden at el. retrospectively evaluated a novel metric utilizing the PK properties of both NPs and matching small molecule payloads in xenograft-bearing mice and syngenetic tumors: the relative distribution over time (RDI-OT) [25,170]. This metric is calculated by taking the tumor drug concentration and dividing by the plasma drug concentration at each time point of note (Figure 4) [25,170]. Prior methods traditionally focused on measuring and equating a ratio of the AUC of overall tissue and plasma exposures, but there had been no effort to try and combine the two measures at individual longitudinal time points to provide analysis of tumor delivery over time as a PK analysis. The AUC of the RDI-OT parameter was a key parameter that was used to determine the efficiency of delivery for the NP being studied [25]. Using standard AUC values for all tissues and plasma was higher for the NPs when compared to the SM drugs (387-fold greater in plasma and 25-fold greater in the tumor) [25]. When evaluating the novel RDI-OT AUC_0-last_ for tumor concentrations against these ‘traditional’ evaluations, 8 out of the 17 (48%) small molecule drugs had higher RDI-OT AUCs compared to their NP formulations [25]. This trend was similar when comparing the tumor RDI-OT AUC_0–6h_, as all small molecule drugs had higher AUCs than the comparator NPs [25,170]. These results show that about half of the small molecule drugs are able to be delivered into the tumor more efficiently over the course of the treatment period in mice bearing flank tumor xenografts. The fact that all the small molecule drugs had higher values than the NPs at the RDI-OT AUC_0–6h_ value also shows that directly after administration, all small molecule drugs are being delivered more efficiently in mice bearing flank tumor xenografts. While these results seem contradictory to the theories surrounding selective NP delivery within tumors, it demonstrates that NPs may actually not accumulate within tumors, but rather provide a slow release of their small-molecule payload in circulation which can readily distribute into tumor [25]. If this mechanism is correct, RDI-OT values would be lower for NPs not due to a lack of delivery efficiency, but rather a previously unknown NP release mechanism primarily reliant on release kinetics in circulation, not within the tumor. While this mechanism is novel, it also highlights the need to improve on current analytical techniques to separate individual NP states (i.e., encapsulated/conjugated, released) within blood, tumor, and tissues to accurately define NP disposition. While these analyses have yet to be utilized in a traditional toxicokinetic or safety evaluation within human subjects, the potential for providing additional information, insight into the incidence, and screening of severe toxicities would provide a much-needed new tool for clinical pharmacologists.

**Figure 5:**
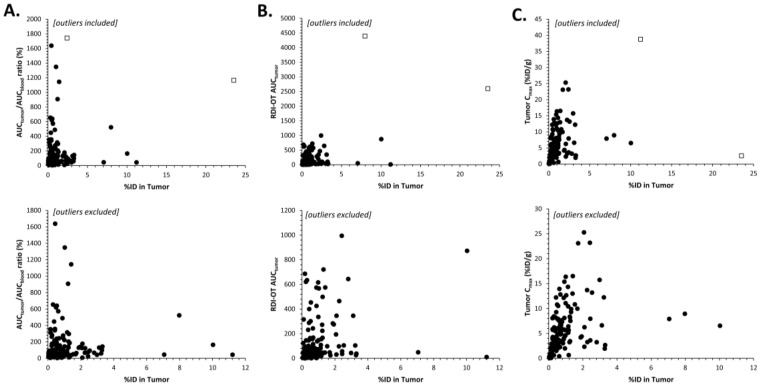
Correlation plots for all data sets between %ID in Tumor (per Wilhelm et al.) and AUCtumor/AUCblood ratio (%) (**A**), RDI-OT AUCtumor (**B**), and tumor Cmax (**C**). Plots are shown with all data sets (outliers shown as □) and with outliers excluded. There was no relationship between %ID in Tumor and AUCtumor/AUCblood ratio (%) [ρ = 0.183 all data (AD); ρ = 0.151 excluding outliers (EO)] and a weak relationship between %ID in Tumor and RDI-OT AUCtumor (ρ = 0.319 AD; ρ = 0.289 EO). There was a moderate relationship between %ID in Tumor and the tumor Cmax (ρ = 0.562 AD; ρ = 0.572 EO). *Reproduced and modified under Creative Commons Attribution NonCommercial License 4.0 (CC BY-NC) from Price* et al. *Sci Adv. 2020;6(29):eaay9249.*

The efficiency of NP drug delivery to solid tumors has been questioned in multiple recent publications. In addition, some of this criticism is based on the use of non-standard PK metrics [172]. To better understand the efficiency and magnitude of tumor delivery by NPs, Price et al. compiled and reanalyzed published murine NP tumor PK data that was used as the source data in the often-cited NP tumor delivery study of Wilhelm et al. [170,172]. Studies included in the Wilhelm et al. analysis that reported matched tumor and blood concentration vs time data (n = 136 of the original 232 datasets) were evaluated using classical PK endpoints, as was the correlation between these traditional PK parameters and the unestablished % injected dose (%ID) in Tumor metric used in the Wilhelm et al. study. The previously utilized % ID in Tumor metric, which attempts to relate tumor exposure to injected dose rather than the relevant systemic exposure, was found to be poorly correlated with standard PK metrics used to describe NP tumor delivery extent (AUC_tumor_/AUC_blood_ ratio), and only moderately associated with maximal tumor concentration (tumor C_max_) (Figure 5). The relative tumor delivery of NPs was determined to be approximately 100-fold greater as assessed by standard AUC_tumor_/AUC_blood_ ratio [median (interquartile range): 76.12% (48.79–158.81)], than by %ID in Tumor [0.67 %ID (0.36–1.19)]. These results strongly suggest that PK metrics and calculations can significantly influence the interpretation of NP tumor delivery and stress the need to first properly support novel PK metrics using traditional approaches.

## 6. Conclusions

Over the past 50 years, drug delivery with NPs has become more advanced and more complex. The solubility and structures of NP are what make them unique and favorable when designing the drug molecule. Due to their uniqueness of each NP, it is difficult to predict the ADME of NP drugs. Preclinical studies don’t directly translate to the human trials, and the disposition, safety and efficacy profiles may be unpredictable due to possible MPS and non-MPS related differences. In addition, this could be due to inappropriate experimental design (such as not including a comparative treatment arm using matched SM agent to the NP for active comparison in safety profiles or not evaluating all PK forms of the NP agent) to allow for appropriate translation into patients.

Although the pharmacology of NPs can be quite unpredictable and complicated, they provide potential advances in the selectivity and specificity in drug delivery. Due to the selectivity and specificity, NPs are continuously being developed and studied in many diseases, such as cancer, infection disease and immune disorders. However, the complex aspects of NPs can only be addressed by both focusing on the design of the formulation and the pharmacology of the NPs. With increased attention being given to actively target NPs to tumors to increase efficacy while minimizing toxicity, the development of both personalized (proving NP based on target moiety) and theragnostic (co-development of a diagnostic NP to predict response/toxicity to the active agent) approaches may provide an objective measure to improving NP delivery, efficacy, and safety [28].

## Figures and Tables

**Table 1:** Summary of FDA-approved carrier-mediated agents (including nanomedicines) with oncology indications for therapeutic use.

**Traditional Nanomedicines**		
**Generic Name**	**Brand Name**	**Approval Date**
Liposomal doxorubicin	Doxil	1995-11-17
Liposomal daunorubicin *(discontinued)*	DaunoXome	1996-04-08
Liposomal cytarabine *(discontinued)*	DepoCyt	1999-08-01
Nab-paclitaxel	Abraxane	2005-01-07
Liposomal vincristine sulfate	Marqibo	2012-08-09
Liposomal irinotecan	Onivyde	2015-10-22
Liposomal cytarabine & daunorubicin	Vyxeos	2017-08-23
**Therapeutic Conjugates**		
**Generic Name**	**Brand Name**	**Approval Date**
Pegfilgrastim	Neulasta	2002-01-31
Pegaspariginase	Oncaspar	2006-07-24
**Antibody-Drug Conjugates**		
**Generic Name**	**Brand Name**	**Approval Date**
Brentuximab vedotin	Adcetris	2011-08-19
Ado-trastuzumab emtansine	Kadcyla	2013-02-22
Inotuzumab ozogamicin	Besponsa	2017-08-17
Gemtuzumab ozogamicin	Mylotarg	2017-09-01
Moxetumomab pasudotox-tdfk	Lumoxiti	2018-09-23
Polatuzumab vedotin	Polivy	2019-06-10
Enfortumab vedotin	Padcev	2019-12-18
Trastuzumab deruxtecan	Enhertu	2019-12-20
Sacituzumab govitecan	Trodelvy	2020-04-22
Belantamab mafodotin-blmf	Blenrep	2020-08-05

## Data Availability

Not applicable.

## References

[1] Li Z., Tan S., Li S., Shen Q., Wang K. (2017). Cancer drug delivery in the nano era: An overview and perspectives (Review). Oncol. Rep..

[2] McClements D.J. (2018). Encapsulation, protection, and delivery of bioactive proteins and peptides using nanoparticle and microparticle systems: A review. Adv. Colloid Interface Sci..

[3] Qi B., Wang C., Ding J., Tao W. (2019). Editorial: Applications of Nanobiotechnology in Pharmacology. Front. Pharmacol..

[4] Pitt G.G., Gratzl M.M., Kimmel G.L., Surles J., Sohindler A. (1981). Aliphatic polyesters II. The degradation of poly (dl-lactide), poly (ε-caprolactone), and their copolymers In Vivo. Biomateials.

[5] Bobo D., Robinson K.J., Islam J., Thurecht K.J., Corrie S.R. (2016). Nanoparticle-Based Medicines: A Review of FDA-Approved Materials and Clinical Trials to Date. Pharm. Res..

[6] Prabhakar U., Maeda H., Jain R.K., Sevick-Muraca E.M., Zamboni W., Farokhzad O.C., Barry S.T., Gabizon A., Grodzinski P., Blakey D.C. (2013). Challenges and Key Considerations of the Enhanced Permeability and Retention Effect for Nanomedicine Drug Delivery in Oncology. Cancer Res..

[7] Zamboni W.C., Torchilin V., Patri A.K., Hrkach J., Stern S., Lee R., Nel A., Panaro N.J., Grodzinski P. (2012). Best Practices in Cancer Nanotechnology: Perspective from NCI Nanotechnology Alliance. Clin. Cancer Res. Off. J. Am. Assoc. Cancer Res..

[8] Caron W.P., Song G., Kumar P., Rawal S., Zamboni W.C. (2012). Interpatient Pharmacokinetic and Pharmacodynamic Variability of Carrier-Mediated Anticancer Agents. Clin. Pharmacol. Ther..

[9] Hoshyar N., Gray S., Han H., Bao G. (2016). The effect of nanoparticle size on in vivo pharmacokinetics and cellular interaction. Nanomedicine.

[10] Starling B.R., Kumar P., Lucas A.T., Barrow D., Farnan L., Hendrix L., Giovinazzo H., Song G., Gehrig P., Bensen J.T. (2019). Mononuclear phagocyte system function and nanoparticle pharmacology in obese and normal weight ovarian and endometrial cancer patients. Cancer Chemother. Pharmacol..

[11] Hamidi M., Azadi A., Rafiei P., Ashrafi H. (2013). A pharmacokinetic overview of nanotechnology-based drug delivery systems: An ADME-oriented approach. Crit. Rev. Ther. Drug.

[12] Malaviya P., Shukal D., Vasavada A.R. (2019). Nanotechnology-based Drug Delivery, Metabolism and Toxicity. Curr. Drug Metab..

[13] Moss D.M., Siccardi M. (2014). Optimizing nanomedicine pharmacokinetics using physiologically based pharmacokinetics modelling. Br. J. Pharmacol..

[14] Zolnik B.S., Sadrieh N. (2009). Regulatory perspective on the importance of ADME assessment of nanoscale material containing drugs. Adv. Drug Deliv. Rev..

[15] Lucas A.T., Madden A.J., Zamboni W.C. (2015). Formulation and physiologic factors affecting the pharmacology of carrier-mediated anticancer agents. Expert Opin. Drug Metab. Toxicol..

[16] Li M., Zou P., Tyner K., Lee S. (2017). Physiologically Based Pharmacokinetic (PBPK) Modeling of Pharmaceutical Nanoparticles. AAPS J..

[17] Rajoli R.K.R., Muttil P., Kunda N. (2020). Pharmacokinetic Modelling to Study the Biodistribution of Nanoparticles. Mucosal Delivery of Drugs and Biologics in Nanoparticles.

[18] Missaoui W.N., Arnold R.D., Cummings B.S. (2018). Toxicological status of nanoparticles: What we know and what we don’t know. Chem. Biol. Interactions.

[19] Chalouni C., Doll S. (2018). Fate of Antibody-Drug Conjugates in Cancer Cells. J. Exp. Clin. Cancer Res..

[20] Zhao L., Ji P., Li Z., Roy P., Sahajwalla C.G. (2013). The Antibody Drug Absorption Following Subcutaneous or Intramuscular Administration and Its Mathematical Description by Coupling Physiologically Based Absorption Process with the Conventional Compartment Pharmacokinetic Model. J. Clin. Pharmacol..

[21] Zolnik B.S., González-Fernández Á., Sadrieh N., Dobrovolskaia M.A. (2010). Nanoparticles and the Immune System. Endocrinology.

[22] Lucas A.T., Madden A.J., Zamboni W.C. (2016). Challenges in preclinical to clinical translation for anticancer carrier-mediated agents. Wiley Interdiscip. Rev. Nanomed. Nanobiotechnol..

[23] Lucas A.T., Price L.S., Schorzman A., Zamboni W.C. (2017). Complex effects of tumor microenvironment on the tumor disposition of carrier-mediated agents. Nanomedicine.

[24] Zamboni W.C., Szebeni J., Kozlov S.V., Lucas A.T., Piscitelli J.A., Dobrovolskaia M.A. (2018). Animal models for analysis of immunological responses to nanomaterials: Challenges and considerations. Adv. Drug Deliv. Rev..

[25] Madden A.J., Rawal S., Sandison K., Schell R., Schorzman A., Deal A., Feng L., Ma P., Mumper R., DeSimone J. (2014). Evaluation of the efficiency of tumor and tissue delivery of carrier-mediated agents (CMA) and small molecule (SM) agents in mice using a novel pharmacokinetic (PK) metric: Relative distribution index over time (RDI-OT). J. Nanoparticle Res..

[26] Schorzman A.N., Lucas A.T., Kagel J.R., Zamboni W.C., Sirianni R., Behkam B. (2018). Methods and Study Designs for Characterizing the Pharmacokinetics and Pharmacodynamics of Carrier-Mediated Agents. Targeted Drug Delivery.

[27] Song G., Darr D.B., Santos C.M., Ross M., Valdivia A., Jordan J.L., Midkiff B.R., Cohen S., Nikolaishvili-Feinberg N., Miller C.R. (2014). Effects of Tumor Microenvironment Heterogeneity on Nanoparticle Disposition and Efficacy in Breast Cancer Tumor Models. Clin. Cancer Res..

[28] Golombek S.K., May J.-N., Theek B., Appold L., Drude N., Kiessling F., Lammers T. (2018). Tumor targeting via EPR: Strategies to enhance patient responses. Adv. Drug Deliv. Rev..

[29] Wu H., Ramanathan R.K., Zamboni B.A., Strychor S., Ramalingam S., Edwards R.P., Friedland D.M., Stoller R.G., Belani C.P., Maruca L.J. (2012). Population Pharmacokinetics of Pegylated Liposomal CKD-602 (S-CKD602) in Patients With Advanced Malignancies. J. Clin. Pharmacol..

[30] Zamboni W.C., Ramalingam S., Friedland D.M., Edwards R.P., Stoller R.G., Strychor S., Maruca L., Zamboni B.A., Belani C.P., Ramanathan R.K. (2009). Phase I and Pharmacokinetic Study of Pegylated Liposomal CKD-602 in Patients with Advanced Malignancies. Clin. Cancer Res..

[31] Salmaso S., Caliceti P. (2013). Stealth Properties to Improve Therapeutic Efficacy of Drug Nanocarriers. J. Drug Deliv..

[32] Jain R.K., Stylianopoulos T. (2010). Delivering nanomedicine to solid tumors. Nat. Rev. Clin. Oncol..

[33] Ruoslahti E. (2002). Specialization of tumour vasculature. Nat. Rev. Cancer.

[34] Greish K. (2007). Enhanced permeability and retention of macromolecular drugs in solid tumors: A royal gate for targeted anticancer nanomedicines. J. Drug Target..

[35] Greish K. (2010). Enhanced permeability and retention (EPR) effect for anticancer nanomedicine drug targeting. Methods Mol. Biol..

[36] Kalyane D., Raval N., Maheshwari R., Tambe V., Kalia K., Tekade R.K. (2019). Employment of enhanced permeability and retention effect (EPR): Nanoparticle-based precision tools for targeting of therapeutic and diagnostic agent in cancer. Mater. Sci. Eng. C Mater. Biol. Appl..

[37] Byrne J.D., Betancourt T., Brannon-Peppas L. (2008). Active targeting schemes for nanoparticle systems in cancer therapeutics. Adv. Drug Deliv. Rev..

[38] Pérez-Herrero E., Fernández-Medarde A. (2015). Advanced targeted therapies in cancer: Drug nanocarriers, the future of chemotherapy. Eur. J. Pharm. Biopharm..

[39] Charrois G.J., Allen T.M. (2004). Drug release rate influences the pharmacokinetics, biodistribution, therapeutic activity, and toxicity of pegylated liposomal doxorubicin formulations in murine breast cancer. Biochim. Biophys. Acta.

[40] Maeda H. (2012). Vascular permeability in cancer and infection as related to macromolecular drug delivery, with emphasis on the EPR effect for tumor-selective drug targeting. Proc. Jpn. Acad. Ser. B Phys. Biol. Sci..

[41] Dobrovolskaia M.A., Patri A.K., Zheng J., Clogston J.D., Ayub N., Aggarwal P., Neun B.W., Hall J.B., McNeil S.E. (2009). Interaction of colloidal gold nanoparticles with human blood: Effects on particle size and analysis of plasma protein binding profiles. Nanomed. NBM.

[42] França A., Aggarwal P., Barsov E.V., Kozlov S.V., Dobrovolskaia M.A., González-Fernández Á. (2011). Macrophage scavenger receptor A mediates the uptake of gold colloids by macrophages in vitro. Nanomedicine.

[43] Fam S.Y., Chee C.F., Yong C.Y., Ho K.L., Mariatulqabtiah A.R., Tan W.S. (2020). Stealth Coating of Nanoparticles in Drug-Delivery Systems. Nanomaterials.

[44] Vonarbourg A., Passirani C., Saulnier P., Benoit J.-P. (2006). Parameters influencing the stealthiness of colloidal drug delivery systems. Biomaterials.

[45] Chao Y., Karmali P.P., Simberg D. (2012). Role of Carbohydrate Receptors in the Macrophage Uptake of Dextran-Coated Iron Oxide Nanoparticles. Adv. Exp. Med. Biol..

[46] Geiser M. (2010). Update on Macrophage Clearance of Inhaled Micro- and Nanoparticles. J. Aerosol Med. Pulm. Drug Deliv..

[47] Platt N., Haworth R., Darley L., Gordon S. (2002). The Many Roles of the Class A Macrophage Scavenger Receptor. Int. Rev. Cytol..

[48] Gough P.J., Gordon S. (2002). The role of scavenger receptors in the innate immune system. Microbes Infect..

[49] Guo C., Yi H., Yu X., Hu F., Zuo D., Subjeck J.R., Wang X.-Y. (2012). Absence of scavenger receptor A promotes dendritic cell-mediated cross-presentation of cell-associated antigen and antitumor immune response. Immunol. Cell Biol..

[50] Pagare P.P., Zaidi S.A., Zhang X., Li X., Yu X., Wang X.-Y., Zhang Y. (2017). Understanding molecular interactions between scavenger receptor A and its natural product inhibitors through molecular modeling studies. J. Mol. Graph. Model..

[51] Patten D., Shetty S. (2018). More Than Just a Removal Service: Scavenger Receptors in Leukocyte Trafficking. Front. Immunol..

[52] Allen L.-A.H., Aderem A. (1996). Mechanisms of phagocytosis. Curr. Opin. Immunol..

[53] Gray M., Botelho R.J. (2017). Phagocytosis: Hungry, Hungry Cells. Methods Mol. Biol..

[54] Swanson J.A. (2008). Shaping cups into phagosomes and macropinosomes. Nat. Rev. Mol. Cell Biol..

[55] Tjelle T.E., Lovdal T., Berg T. (2000). Phagosome dynamics and function. BioEssays.

[56] Chen H., Langer R., Edwards D.A. (1997). A Film Tension Theory of Phagocytosis. J. Colloid Interface Sci..

[57] Davda J., Labhasetwar V. (2002). Characterization of nanoparticle uptake by endothelial cells. Int. J. Pharm..

[58] Panyam J., Labhasetwar V. (2003). Dynamics of Endocytosis and Exocytosis of Poly(D,L-Lactide-co-Glycolide) Nanoparticles in Vascular Smooth Muscle Cells. Pharm. Res..

[59] Kamps J.A., Scherphof G.L. (1998). Receptor versus non-receptor mediated clearance of liposomes. Adv. Drug Deliv. Rev..

[60] Patel H.M. (1992). Serum opsonins and liposomes: Their interaction and opsonophagocytosis. Crit. Rev. Ther. Drug Carr. Syst..

[61] Patel H., Moghimi S.M. (1998). Serum-mediated recognition of liposomes by phagocytic cells of the reticuloendothelial system—The concept of tissue specificity. Adv. Drug Deliv. Rev..

[62] Brown M.S., Goldstein J.L. (1983). Lipoprotein Metabolism in the Macrophage: Implications for Cholesterol Deposition in Atherosclerosis. Annu. Rev. Biochem..

[63] Linehan S.A., Martinez-Pomares L., Gordon S. (2000). Mannose Receptor and Scavenger Receptor: Two Macrophage Pattern Recognition Receptors with Diverse Functions in Tissue Homeostasis and Host Defense. Adv. Exp. Med. Biol..

[64] Chnari E., Nikitczuk J.S., Wang J., Uhrich K.E., Moghe P.V. (2006). Engineered Polymeric Nanoparticles for Receptor-Targeted Blockage of Oxidized Low Density Lipoprotein Uptake and Atherogenesis in Macrophages. Biomacromolecules.

[65] Kamps J.A., Morselt H.W., Scherphof G.L. (1999). Uptake of Liposomes Containing Phosphatidylserine by Liver Cells In Vivo and by Sinusoidal Liver Cells in Primary Culture: In Vivo–In Vitro Differences. Biochem. Biophys. Res. Commun..

[66] Moghimi S.M., Hunter A.C. (2001). Recognition by macrophages and liver cells of opsonized phospholipid vesicles and phospholipid headgroups. Pharm. Res..

[67] Liu D., Hu Q., Song Y.K. (1995). Liposome clearance from blood: Different animal species have different mechanisms. Biochim. Biophys. Acta.

[68] Liu F., Liu D. (1996). Serum independent liposome uptake by mouse liver. Biochim. Biophys. Acta.

[69] Merle N.S., Church S.E., Fremeaux-Bacchi V., Roumenina L.T. (2015). Complement System Part I—Molecular Mechanisms of Activation and Regulation. Front. Immunol..

[70] Merle N.S., Noe R., Halbwachs-Mecarelli L., Fremeaux-Bacchi V., Roumenina L.T. (2015). Complement System Part II: Role in Immunity. Front. Immunol..

[71] Noris M., Remuzzi G. (2013). Overview of Complement Activation and Regulation. Semin. Nephrol..

[72] Szebeni J. (2001). Complement Activation-Related Pseudoallergy Caused by Liposomes, Micellar Carriers of Intravenous Drugs, and Radiocontrast Agents. Crit. Rev. Ther. Drug Carr. Syst..

[73] Szebeni J. (2014). Complement activation-related pseudoallergy: A stress reaction in blood triggered by nanomedicines and biologicals. Mol. Immunol..

[74] Szebeni J., Bedőcs P., Rozsnyay Z., Weiszhár Z., Urbanics R., Rosivall L., Cohen R., Garbuzenko O., Báthori G., Tóth M. (2012). Liposome-induced complement activation and related cardiopulmonary distress in pigs: Factors promoting reactogenicity of Doxil and AmBisome. Nanomed. Nanotechnol. Biol. Med..

[75] Haney M.S., Bohlen C.J., Morgens D.W., Ousey J.A., Barkal A.A., Tsui C.K., Ego B.K., Levin R., Kamber R.A., Collins H. (2018). Identification of phagocytosis regulators using magnetic genome-wide CRISPR screens. Nat. Genet..

[76] Martincorena I., Campbell P.J. (2015). Somatic mutation in cancer and normal cells. Science.

[77] Hashizume H., Baluk P., Morikawa S., McLean J.W., Thurston G., Roberge S., Jain R.K., McDonald D.M. (2000). Openings between Defective Endothelial Cells Explain Tumor Vessel Leakiness. Am. J. Pathol..

[78] Hobbs S.K., Monsky W.L., Yuan F., Roberts W.G., Griffith L., Torchilin V.P., Jain R.K. (1998). Regulation of transport pathways in tumor vessels: Role of tumor type and microenvironment. Proc. Natl. Acad. Sci. USA.

[79] Sindhwani S., Syed A.M., Ngai J., Kingston B.R., Maiorino L., Rothschild J., Macmillan P., Zhang Y., Rajesh N.U., Hoang T. (2020). The entry of nanoparticles into solid tumours. Nat. Mater..

[80] Khawar I.A., Kim J.H., Kuh H.-J. (2015). Improving drug delivery to solid tumors: Priming the tumor microenvironment. J. Control. Release.

[81] Miao L., Lin C.M., Huang L. (2015). Stromal barriers and strategies for the delivery of nanomedicine to desmoplastic tumors. J. Control. Release.

[82] Overchuk M., Zheng G. (2018). Overcoming obstacles in the tumor microenvironment: Recent advancements in nanoparticle delivery for cancer theranostics. Biomaterials.

[83] Curtis L.T., Frieboes H.B. (2016). The Tumor Microenvironment as a Barrier to Cancer Nanotherapy. Adv. Exp. Med. Biol..

[84] Goel S., Wong A.H.-K., Jain R.K. (2012). Vascular Normalization as a Therapeutic Strategy for Malignant and Nonmalignant Disease. Cold Spring Harb. Perspect. Med..

[85] Jain R.K. (2005). Normalization of Tumor Vasculature: An Emerging Concept in Antiangiogenic Therapy. Science.

[86] Wang J., Lu Z., Gao Y., Wientjes M.G., Au J.L.-S. (2011). Improving delivery and efficacy of nanomedicines in solid tumors: Role of tumor priming. Nanomedicine.

[87] Yuan F., Dellian M., Fukumura D., Leunig M., Berk D.A., Torchilin V.P., Jain R.K. (1995). Vascular permeability in a human tumor xenograft: Molecular size dependence and cutoff size. Cancer Res..

[88] Tong R.T., Boucher Y., Kozin S.V., Winkler F., Hicklin D.J., Jain R.K. (2004). Vascular Normalization by Vascular Endothelial Growth Factor Receptor 2 Blockade Induces a Pressure Gradient Across the Vasculature and Improves Drug Penetration in Tumors. Cancer Res..

[89] Moghimi S.M., Hunter A.C., Murray J.C. (2001). Long-circulating and target-specific nanoparticles: Theory to practice. Pharmacol. Rev..

[90] Hendry S.A., Farnsworth R.H., Solomon B., Achen M.G., Stacker S.A., Fox S.B. (2016). The Role of the Tumor Vasculature in the Host Immune Response: Implications for Therapeutic Strategies Targeting the Tumor Microenvironment. Front. Immunol..

[91] Kim J.H., Suh J.-Y., Woo D.-C., Sung Y.S., Son W.-C., Choi Y.S., Pae S.J., Kim J.K. (2016). Difference in the intratumoral distributions of extracellular-fluid and intravascular MR contrast agents in glioblastoma growth. NMR Biomed..

[92] Sarin H., Kanevsky A.S., Wu H., Sousa A.A., Wilson C.M., Aronova M.A., Griffiths G.L., Leapman R.D., Vo H.Q. (2009). Physiologic upper limit of pore size in the blood-tumor barrier of malignant solid tumors. J. Transl. Med..

[93] Guan Z., Wang L., Lin J. (2017). Interaction Pathways between Plasma Membrane and Block Copolymer Micelles. Biomacromolecules.

[94] Lee H., Hoang B., Fonge H., Reilly R.M., Allen C. (2010). In Vivo Distribution of Polymeric Nanoparticles at the Whole-Body, Tumor, and Cellular Levels. Pharm. Res..

[95] Zhang J., Liu J., Zhao Y., Wang G., Zhou F. (2015). Plasma and cellular pharmacokinetic considerations for the development and optimization of antitumor block copolymer micelles. Expert Opin. Drug Deliv..

[96] Wang M., Zhao J., Zhang L., Wei F., Lian Y., Wu Y., Gong Z., Zhang S., Zhou J., Cao K. (2017). Role of tumor microenvironment in tumorigenesis. J. Cancer.

[97] Guo S., Deng C. (2018). Effect of Stromal Cells in Tumor Microenvironment on Metastasis Initiation. Int. J. Biol. Sci..

[98] Mitchell M.J., Jain R.K., Langer R. (2017). Engineering and physical sciences in oncology: Challenges and opportunities. Nat. Rev. Cancer.

[99] De Palma M., Biziato D., Petrova T.V. (2017). Microenvironmental regulation of tumour angiogenesis. Nat. Rev. Cancer.

[100] Brown E.B., Boucher Y., Nasser S., Jain R.K. (2004). Measurement of macromolecular diffusion coefficients in human tumors. Microvasc. Res..

[101] Buescher C., Hoo K.A., Janssen H. (2008). An Experimental Approach to Measure Mass Diffusion in Rat Tumor Tissue. IEEE Trans. Biomed. Eng..

[102] Netti P.A., Berk D.A., Swartz M.A., Grodzinsky A.J., Jain R.K. (2000). Role of extracellular matrix assembly in interstitial transport in solid tumors. Cancer Res..

[103] Goodman T.T., Olive P.L., Pun S.H. (2007). Increased nanoparticle penetration in collagenase-treated multicellular spheroids. Int. J. Nanomed..

[104] Goins B., Phillips W.T., Bao A. (2016). Strategies for improving the intratumoral distribution of liposomal drugs in cancer therapy. Expert Opin. Drug Deliv..

[105] Miao L., Huang L. (2015). Exploring the Tumor Microenvironment with Nanoparticles. Cancer Treat. Res..

[106] Hoogsteen I.J., Marres H.A.M., Bussink J., Van Der Kogel A.J., Kaanders J.H.A.M. (2007). Tumor microenvironment in head and neck squamous cell carcinomas: Predictive value and clinical relevance of hypoxic markers. A review. Head Neck.

[107] Ljungkvist A.S.E., Bussink J., Kaanders J.H.A.M., Van Der Kogel A.J. (2007). Dynamics of Tumor Hypoxia Measured with Bioreductive Hypoxic Cell Markers. Radiat. Res..

[108] Ljungkvist A.S.E., Bussink J., Rijken P.F.J.W., Kaanders J.H.A.M., Van Der Kogel A.J., Denekamp J. (2002). Vascular architecture, hypoxia, and proliferation in first-generation xenografts of human head-and-neck squamous cell carcinomas. Int. J. Radiat. Oncol..

[109] Farhood B., Najafi M., Mortezaee K. (2019). Cancer-associated fibroblasts: Secretions, interactions, and therapy. J. Cell. Biochem..

[110] Kakarla S., Song X.-T., Gottschalk S. (2012). Cancer-associated fibroblasts as targets for immunotherapy. Immunotherapy.

[111] Räsänen K., Vaheri A. (2010). Activation of fibroblasts in cancer stroma. Exp. Cell Res..

[112] Truffi M., Mazzucchelli S., Bonizzi A., Sorrentino L., Allevi R., Vanna R., Morasso C., Corsi F. (2019). Nano-Strategies to Target Breast Cancer-Associated Fibroblasts: Rearranging the Tumor Microenvironment to Achieve Antitumor Efficacy. Int. J. Mol. Sci..

[113] Zhang Y., Ho S., Li B., Nie G., Li S. (2020). Modulating the tumor microenvironment with new therapeutic nanoparticles: A promising paradigm for tumor treatment. Med. Res. Rev..

[114] Murakami M., Ernsting M.J., Undzys E., Holwell N., Foltz W.D., Li S.-D. (2013). Docetaxel Conjugate Nanoparticles That Target α-Smooth Muscle Actin–Expressing Stromal Cells Suppress Breast Cancer Metastasis. Cancer Res..

[115] Miao L., Liu Q., Lin C.M., Luo C., Wang Y., Liu L., Yin W., Hu S., Kim W.Y., Huang L. (2017). Targeting Tumor-Associated Fibroblasts for Therapeutic Delivery in Desmoplastic Tumors. Cancer Res..

[116] Heldin C.-H., Rubin K., Pietras K., Östman A. (2004). High interstitial fluid pressure — an obstacle in cancer therapy. Nat. Rev. Cancer.

[117] Rippe B., Haraldsson B. (1994). Transport of macromolecules across microvascular walls: The two-pore theory. Physiol. Rev..

[118] Baronzio G., Parmar G., Baronzio M. (2015). Overview of Methods for Overcoming Hindrance to Drug Delivery to Tumors, with Special Attention to Tumor Interstitial Fluid. Front. Oncol..

[119] Stapleton S., Milosevic M., Tannock I.F., Allen C., Jaffray D.A. (2015). The intra-tumoral relationship between microcirculation, interstitial fluid pressure and liposome accumulation. J. Control. Release.

[120] Zhao Y., Cao J., Melamed A., Worley M., Gockley A., Jones D., Nia H.T., Zhang Y., Stylianopoulos T., Kumar A.S. (2019). Losartan treatment enhances chemotherapy efficacy and reduces ascites in ovarian cancer models by normalizing the tumor stroma. Proc. Natl. Acad. Sci. USA.

[121] Zamboni W.C., Strychor S., Joseph E., Walsh D.R., Zamboni B.A., Parise R.A., Tonda M.E., Yu N.Y., Engbers C., Eiseman J.L. (2007). Plasma, Tumor, and Tissue Disposition of STEALTH Liposomal CKD-602 (S-CKD602) and Nonliposomal CKD-602 in Mice Bearing A375 Human Melanoma Xenografts. Clin. Cancer Res..

[122] O’Neal S.K., Lucas A.T., Caron W.P., Song G., Lay J.C., Zamboni W.C., Dobrovolskaia M.A., McNeil S.E. (2016). Bidirectional Interaction between Nanoparticles and Carrier-Mediated Agents and Cells of the Mononuclear Phagocytic System. Handbook of Immunological Properties of Engineered Nanomaterials.

[123] Lucas A.T., White T.F., Deal A.M., Herity L.B., Song G., Santos C.M., Zamboni W.C. (2017). Profiling the relationship between tumor-associated macrophages and pharmacokinetics of liposomal agents in preclinical murine models. Nanomed. Nanotechnol. Biol. Med..

[124] Alizadeh D., Zhang L., Hwang J., Schluep T., Badie B. (2010). Tumor-associated macrophages are predominant carriers of cyclodextrin-based nanoparticles into gliomas. Nanomed. Nanotechnol. Biol. Med..

[125] van Claasen R.H.H., Kluin P.M., Fleuren G.J. (1992). Tumor infiltrating cells in human cancer. On the possible role of CD16+ macrophages in antitumor cytotoxicity. Lab. Investig..

[126] Al-Sarireh B., Eremin O. (2000). Tumour-associated macrophages (TAMS): Disordered function, immune suppression and progressive tumour growth. J. R. Coll. Surg. Edinb..

[127] Kitamura T., Qian B.-Z., Pollard J.W. (2015). Immune cell promotion of metastasis. Nat. Rev. Immunol..

[128] Mitchem J.B., Brennan D.J., Knolhoff B.L., Belt B.A., Zhu Y., Sanford D.E., Belaygorod L., Carpenter D., Collins L., Piwnica-Worms D. (2013). Targeting tumor-infiltrating macrophages decreases tumor-initiating cells, relieves immunosuppression, and improves chemotherapeutic responses. Cancer Res..

[129] Shaffer S.A., Baker-Lee C., Kennedy J., Lai M.S., De Vries P., Buhler K., Singer J.W. (2007). In Vitro and In Vivo metabolism of paclitaxel poliglumex: Identification of metabolites and active proteases. Cancer Chemother. Pharmacol..

[130] Jackson E.F., Esparza-Coss E., Wen X., Ng C.S., Daniel S.L., Price R.E., Rivera B., Charnsangavej C., Gelovani J.G., Li C. (2007). Magnetic Resonance Imaging of Therapy-Induced Necrosis Using Gadolinium-Chelated Polyglutamic Acids. Int. J. Radiat. Oncol..

[131] Rafiyath S.M., Rasul M., Lee B., Wei G., Lamba G., Liu D. (2012). Comparison of safety and toxicity of liposomal doxorubicin vs. conventional anthracyclines: A meta-analysis. Exp. Hematol. Oncol..

[132] Senapati S., Mahanta A.K., Kumar S., Maiti P. (2018). Controlled drug delivery vehicles for cancer treatment and their performance. Signal Transduct. Target. Ther..

[133] Feng X., Li D., Han J., Zhuang X., Ding J. (2017). Schiff base bond-linked polysaccharide–doxorubicin conjugate for upregulated cancer therapy. Mater. Sci. Eng. C Mater. Biol. Appl..

[134] Li D., Han J., Ding J., Chen L., Chen X. (2017). Acid-sensitive dextran prodrug: A higher molecular weight makes a better efficacy. Carbohydr. Polym..

[135] Newman M.S., Colbern G.T., Working P.K., Engbers C., Amantea M.A. (1999). Comparative pharmacokinetics, tissue distribution, and therapeutic effectiveness of cisplatin encapsulated in long-circulating, pegylated liposomes (SPI-077) in tumor-bearing mice. Cancer Chemother. Pharmacol..

[136] Harrington K., Lewanski C.R., Northcote A.D., Whittaker J., Wellbank H., Vile R.G., Peters A.M., Stewart J.S.W. (2001). Phase I–II study of pegylated liposomal cisplatin (SPI-077) in patients with inoperable head and neck cancer. Ann. Oncol: Off. J. Eur. Soc. Med. Oncol..

[137] Seetharamu N., Kim E., Hochster H., Martin F., Muggia F. (2010). Phase II study of liposomal cisplatin (SPI-77) in platinum-sensitive recurrences of ovarian cancer. Anticancer Res..

[138] White S.C., Lorigan P., Margison G.P., Margison J.M., Martin F., Thatcher N., Anderson H., Ranson M. (2006). Phase II study of SPI-77 (sterically stabilised liposomal cisplatin) in advanced non-small-cell lung cancer. Br. J. Cancer.

[139] Terwogt J.M.M., Groenewegen G., Pluim D., Maliepaard M., Tibben M.M., Huisman A., Huinink W.W.T.B., Schot M., Welbank H., Voest E.E. (2002). Phase I and pharmacokinetic study of SPI-77, a liposomal encapsulated dosage form of cisplatin. Cancer Chemother. Pharmacol..

[140] Zamboni W.C., Gervais A.C., Egorin M.J., Schellens J.H.M., Zuhowski E.G., Pluim D., Joseph E., Hamburger D.R., Working P.K., Colbern G. (2004). Systemic and tumor disposition of platinum after administration of cisplatin or STEALTH liposomal-cisplatin formulations (SPI-077 and SPI-077 B103) in a preclinical tumor model of melanoma. Cancer Chemother. Pharmacol..

[141] Brown E., McKee T., diTomaso E., Pluen A., Seed B., Boucher Y., Jain R.K. (2003). Dynamic imaging of collagen and its modulation in tumors in vivo using second-harmonic generation. Nat. Med..

[142] Choi J., Credit K., Henderson K., Deverkadra R., He Z., Wiig H., Vanpelt H., Flessner M.F. (2006). Intraperitoneal Immunotherapy for Metastatic Ovarian Carcinoma: Resistance of Intratumoral Collagen to Antibody Penetration. Clin. Cancer Res. Off. J. Am. Assoc. Cancer Res..

[143] Perentes J.Y., McKee T.D., Ley C.D., Mathiew H., Dawson M., Padera T.P., Munn L.L., Jain R.K., Boucher Y. (2009). In Vivo imaging of extracellular matrix remodeling by tumor-associated fibroblasts. Nat. Methods.

[144] Chauhan V.P., Martin J.D., Liu H., Lacorre D.A., Jain S.R., Kozin S.V., Stylianopoulos T., Mousa A.S., Han X., Adstamongkonkul P. (2013). Angiotensin inhibition enhances drug delivery and potentiates chemotherapy by decompressing tumour blood vessels. Nat. Commun..

[145] Davies C.D.L., Lundstrøm L.M., Frengen J., Eikenes L., Bruland Ø.S., Kaalhus O., Hjelstuen M.H.B., Brekken C. (2004). Radiation Improves the Distribution and Uptake of Liposomal Doxorubicin (Caelyx) in Human Osteosarcoma Xenografts. Cancer Res..

[146] Manzoor A.A., Lindner L.H., Landon C.D., Park J.-Y., Simnick A.J., Dreher M.R., Das S., Hanna G., Park W., Chilkoti A. (2012). Overcoming Limitations in Nanoparticle Drug Delivery: Triggered, Intravascular Release to Improve Drug Penetration into Tumors. Cancer Res..

[147] Needham D., Anyarambhatla G., Kong G., Dewhirst M.W. (2000). A new temperature-sensitive liposome for use with mild hyperthermia: Characterization and testing in a human tumor xenograft model. Cancer Res..

[148] Zhao Y., Alakhova D.Y., Kim J.O., Bronich T.K., Kabanov A.V. (2013). A simple way to enhance Doxil® therapy: Drug release from liposomes at the tumor site by amphiphilic block copolymer. J. Control. Release.

[149] La-Beck N.M., Liu X., Wood L.M. (2019). Harnessing Liposome Interactions With the Immune System for the Next Breakthrough in Cancer Drug Delivery. Front. Pharmacol..

[150] Gabrilovich D.I., Nagaraj S. (2009). Myeloid-derived suppressor cells as regulators of the immune system. Nat. Rev. Immunol..

[151] Lasek W., Zagożdżon R., Jakobisiak M. (2014). Interleukin 12: Still a promising candidate for tumor immunotherapy?. Cancer Immunol. Immunother..

[152] Mantovani A., Sozzani S., Locati M., Allavena P., Sica A. (2002). Macrophage polarization: Tumor-associated macrophages as a paradigm for polarized M2 mononuclear phagocytes. Trends Immunol..

[153] Rajan R., Sabnani M.K., Mavinkurve V., Shmeeda H., Mansouri H., Bonkoungou S., Le A.D., Wood L.M., Gabizon A.A., La-Beck N.M. (2018). Liposome-induced immunosuppression and tumor growth is mediated by macrophages and mitigated by liposome-encapsulated alendronate. J. Control. Release.

[154] Allavena P., Sica A., Solinas G., Porta C., Mantovani A. (2008). The inflammatory micro-environment in tumor progression: The role of tumor-associated macrophages. Crit. Rev. Oncol. Hematol..

[155] Otsuka M., Tsuchiya S., Aramaki Y. (2004). Involvement of ERK, a MAP kinase, in the production of TGF-beta by macrophages treated with liposomes composed of phosphatidylserine. Biochem. Biophys. Res. Commun..

[156] Lucas A.T., Herity L.B., Kornblum Z.A., Madden A.J., Gabizon A., Kabanov A.V., Ajamie R.T., Bender D.M., Kulanthaivel P., Sanchez-Felix M. (2017). Pharmacokinetic and screening studies of the interaction between mononuclear phagocyte system and nanoparticle formulations and colloid forming drugs. Int. J. Pharm..

[157] Caron W.P., Lay J.C., Fong A.M., La-Beck N.M., Kumar P., Newman S.E., Zhou H., Monaco J.H., Clarke-Pearson D.L., Brewster W.R. (2013). Translational Studies of Phenotypic Probes for the Mononuclear Phagocyte System and Liposomal Pharmacology. J. Pharmacol. Exp. Ther..

[158] Falandry C., Brain E., Bonnefoy M., Mefti F., Jovenin N., Rigal O., Guillem O., El Kouri C., Uwer L., Abadie-Lacourtoisie S. (2013). Impact of geriatric risk factors on pegylated liposomal doxorubicin tolerance and efficacy in elderly metastatic breast cancer patients: Final results of the DOGMES multicentre GINECO trial. Eur. J. Cancer.

[159] Sostelly A., Henin E., Chauvenet L., Hardy-Bessard A.-C., Tallec V.J.-L., Kirsher S., Leyronnas C., Ligeza-Poisson C., Ramdane S., Salavt J. (2013). Can we predict chemo-induced hematotoxicity in elderly patients treated with pegylated liposomal doxorubicin? Results of a population-based model derived from the DOGMES phase II trial of the GINECO. J. Geriatr. Oncol..

[160] Grobner T. (2006). Gadolinium—A specific trigger for the development of nephrogenic fibrosing dermopathy and nephrogenic systemic fibrosis?. Nephrol. Dial. Transplant..

[161] Xiao Y.-D., Paudel R., Liu J., Ma C., Zhang Z.-S., Zhou S.-K. (2016). MRI contrast agents: Classification and application (Review). Int. J. Mol. Med..

[162] Swaminathan S. (2016). Gadolinium toxicity: Iron and ferroportin as central targets. Magn. Reson. Imaging.

[163] Miyamoto J., Tanikawa A., Igarashi A., Hataya H., Kobayashi K., Ikegami M., Sotome A., Nagai Y., Kameyama K., Ishiko A. (2011). Detection of Iron Deposition in Dermal Fibrocytes Is a Useful Tool for Histologic Diagnosis of Nephrogenic Systemic Fibrosis. Am. J. Dermatopathol..

[164] Song G., Tarrant T.K., White T.F., Barrow D.A., Santos C.M., Timoshchenko R.G., Hanna S.K., Ramanathan R.K., Lee C.R., Bae-Jump V. (2015). Roles of chemokines CCL2 and CCL5 in the pharmacokinetics of PEGylated liposomal doxorubicin in vivo and in patients with recurrent epithelial ovarian cancer. Nanomed. Nanotechnol. Biol. Med..

[165] Felici A., Verweij J., Sparreboom A. (2002). Dosing strategies for anticancer drugs: The good, the bad and body-surface area. Eur. J. Cancer.

[166] Mahmood I. (1996). Interspecies scaling: Predicting clearance of anticancer drugs in humans. A comparative study of three different approaches using body weight or body surface area. Eur. J. Drug Metab. Pharmacokinet..

[167] Sahneh F.D., Scoglio C.M., Monteiro-Riviere N.A., Riviere J.E. (2015). Predicting the impact of biocorona formation kinetics on interspecies extrapolations of nanoparticle biodistribution modeling. Nanomedicine.

[168] Caron W.P., Clewell H., Dedrick R., Ramanathan R.K., Davis W.L., Yu N., Tonda M., Schellens J.H., Beijnen J.H., Zamboni W.C. (2011). Allometric scaling of pegylated liposomal anticancer drugs. J. Pharmacokinet. Pharmacodyn..

[169] Caron W.P., Morgan K.P., Zamboni B.A., Zamboni W.C. (2013). A Review of Study Designs and Outcomes of Phase I Clinical Studies of Nanoparticle Agents Compared with Small-Molecule Anticancer Agents. Clin. Cancer Res..

[170] Price L.S.L., Stern S.T., Deal A.M., Kabanov A.V., Zamboni W.C. (2020). A reanalysis of nanoparticle tumor delivery using classical pharmacokinetic metrics. Sci. Adv..

[171] Schell R.F., Sidone B.J., Caron W.P., Walsh M.D., White T.F., Zamboni B.A., Ramanathan R.K., Zamboni W.C. (2014). Meta-analysis of inter-patient pharmacokinetic variability of liposomal and non-liposomal anticancer agents. Nanomed. Nanotechnol. Biol. Med..

[172] Wilhelm S., Tavares A.J., Dai Q., Ohta S., Audet J., Dvorak H.F., Chan W.C.W. (2016). Analysis of nanoparticle delivery to tumours. Nat. Rev. Mater..

